# Functionalized Fiber-Based Strain Sensors: Pathway to Next-Generation Wearable Electronics

**DOI:** 10.1007/s40820-022-00806-8

**Published:** 2022-02-15

**Authors:** Zekun Liu, Tianxue Zhu, Junru Wang, Zijian Zheng, Yi Li, Jiashen Li, Yuekun Lai

**Affiliations:** 1 Department of Materials, The University of Manchester, Oxford Road, Manchester, M13 9PL UK; 2 College of Chemical Engineering, Fuzhou University, Fuzhou, 350116 China; 3 Institute of Textiles and Clothing, Research Institute for Intelligent Wearable Systems, Research Institute for Smart Energy, The Hong Kong Polytechnic University, Hung Hom, Hong Kong, SAR China

**Keywords:** Wearable strain sensor, Fiber functionalization, Wearability, Flexible electronics, Conductive materials

## Abstract

Wearable strain sensors are arousing increasing research interests in recent years on account of their potentials in motion detection, personal and public healthcare, future entertainment, man–machine interaction, artificial intelligence, and so forth. Much research has focused on fiber-based sensors due to the appealing performance of fibers, including processing flexibility, wearing comfortability, outstanding lifetime and serviceability, low-cost and large-scale capacity. Herein, we review the latest advances in functionalization and device fabrication of fiber materials toward applications in fiber-based wearable strain sensors. We describe the approaches for preparing conductive fibers such as spinning, surface modification, and structural transformation. We also introduce the fabrication and sensing mechanisms of state-of-the-art sensors and analyze their merits and demerits. The applications toward motion detection, healthcare, man–machine interaction, future entertainment, and multifunctional sensing are summarized with typical examples. We finally critically analyze tough challenges and future remarks of fiber-based strain sensors, aiming to implement them in real applications.

## Highlights

- General principles for fiber functionalization and strain sensor fabrication are briefly reviewed.
- Future application potentials of wearable strain sensors are summarized and evaluated.
- Challenges and perspectives of fiber-based wearable strain sensors are critically discussed.

## Graphical abstract

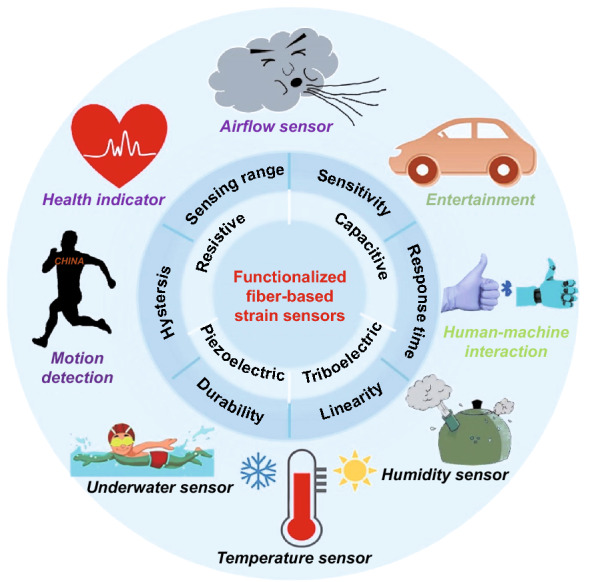

## Introduction

Real-time physical perception by stretchable and skin-mountable strain sensors plays a vital role in understanding the applications of personal and public healthcare management, artificial intelligence, and future fictitious entertainment [1–11]. Such devices can immediately and directly convert physical stimulation into gaugeable signals with the advantages of flexibility, wearability, lightweight, high efficiency, and accuracy [12–18]. They overcome the limited sensing range and non-portability of conventional rigid sensors, driving a technological revolution in next-generation electronics [19–26].

Several materials have illustrated their potentials in stretchable strain sensors, and fiber is a competitive candidate. Fibers have been widely used for human clothes and other commodities in daily life for thousands of years. Mankind benefits from them by utilizing their outstanding features, e.g., the excellent resilience and durability of hemp, remarkable skin-touch and structure controllability of silk, as well as the outstanding thermal protection, elasticity, moisture absorption, and excellent perspiration conductivity of cotton [27–35]. In recent years, it has been found that they are also very competitive as substrate materials for wearable electronics on account of both outstanding flexibility and permeability, as well as validated harmlessness to the human body [36–46]. These flexible substrates can be developed as wearable electronics via various processing: carbonization, graphitization, printing, chemical/electrolytic deposition, graft copolymerization, and so forth [47–57]. Besides, some synthetic fibers made with graphene, carbon nanotube (CNT), metals, and conductive polymers endow them with good conductivity and flexibility in nature [58–62]. The attributes pave a new way for the low-cost and scale fabrication of wearable strain sensors.

With the above-mentioned superiorities of wearable strain sensors based on fiber materials, this review aims to comprehensively survey skin-mountable and fiber-based wearable strain sensors (FWSSs) in materials preparation, device manufacture, performance evolution, mechanism discussion, and application potential. The conductive fiber preparation approaches including spinning, surface modification, and structural transformation are reviewed. The device manufactures are described and classified according to whether the encapsulated layer’s existence i.e., encapsulated and encapsulation-free strain sensors, including general approach (elastomer encapsulating conductive networks) and textile forming technology. The advantages and disadvantages between them are also critically analyzed. The sensing mechanism and future applications related to strain-responsive sensors are explained and evaluated. It shows that the fiber-based sensors enable integration applications in future electronics with advantages of wide-range sensing ability, high sensitivity, remarkable environment stability, and outstanding wearing feelings. Even though it exists published reviews about wearable strain sensors, this work particularly focuses on fiber functionalization, wearability, and wearing comfort, as well as sensing reliability in the fickle wearable microclimate of the devices. We finally critically evaluate existing gaps and future remarks to implement strain sensors in real applications.

## Classification and Performance

### Classification and Characteristics

There are resistive, capacitive, piezoelectric, triboelectric, and optical FWSSs from a sensing mechanism perspective [63–70]. The resistive and capacitive FWSSs possess uncomplicated fabrication, structure configuration, and signal-collect system, as well as decent flexibility and sensing reliability. By contrast, the piezoelectric, triboelectric FWSSs still face many challenges including poor sensing reliability in static or dynamic conditions [71]. Though previous research has shown optical fibers-based strain sensors with promising results in terms of stretchability and durability [72], such devices face the challenge of poor sensitivity to detect extremely small strains [73], which restricts their application scenarios. Therefore, we will mainly focus on resistive and capacitive FWSSs in this work. The FWSSs are prepared with a layer of fiber-based electrodes. When applied with a certain voltage, the resistance of the fiber electrode changes proportionally with the applied strain. The applied strain on the fiber electrode will cause disconnection or slippage between the conducting materials, reducing the contact area between materials and causing a change in resistance [74–77]. The resistance change can be due to the changes in the geometry (area (A) and length (L)) of the textile upon stretching. Alternatively, it could be from piezoresistive behavior, as changes in resistivity (ρ) of the conductive material used in the textile sensor in Eq. 1.

1 $$R = \frac{{{\rho L}}}{A}$$

Capacitive strain sensors, which are also widely used nowadays, respond to strain by changing capacitance. Different from resistive strain sensors, a typical capacitive strain sensor consists of two fiber electrodes and a dielectric layer, where two electrodes are separated by the dielectric, forming a sandwich structure [78–81]. The parallel-plate structure is typical for a capacitive strain sensor because of its easy preparation and direct model. Upon applying a direct current voltage, opposite charges accumulate on the top and bottom electrodes. The current cannot pass through them directly because of the existence of the dielectric layer, and the whole structure acts as a capacitance. The value of the capacitance (*C*) depends on the overlap area between two electrodes (*A*), the thickness of the dielectric layer (*d*), and the relative permittivity of the dielectric layer (ε_r_), as shown in Eq. 2, where ε_0_ is the permittivity of free space. The capacitance changes obviously with the change of geometrical under loading and unloading and is irrelevant to the resistance of the fiber electrodes. When being stretched, the capacitive sensors maintain the capacitive area and decrease the thickness of the dielectric layer, which leads to an increase in capacitance.

2 $$C = \frac{{\varepsilon {\text{r}}\varepsilon 0A}}{d}$$

### Performance Evaluation

The sensing property of FWSSs can be characterized by several performance parameters such as sensing range, sensitivity, response and recovery time, linearity, hysteresis, and durability. These properties are essential for determining sensing reliability and accuracy in practical applications [76, 82–90]. FWSSs should withstand considerable elongation without significantly reducing mechanical properties. As part of the stretch and recovery process, it is vital to design a fabrication scheme that assures the sensor's electrical conductivity. Sensors should sense the entire range of strains necessary for their intended use. In terms of resistance/capacitance-based strain sensors, the resistance/capacitance change during stretching and recovery process determines the sensitivity (indicated by gauge factor (GF)) of the FWSSs. As shown in Eqs. 3 and 4, the sensitivity is quantified as the ratio of resistance/capacitance to applied strain.

3 $${\text{GF}} = \frac{\Delta R/R0}{\varepsilon }$$

In this equation, *R*_0_ is the resistance of strain sensors in an un-stretched state, where *ΔR* is the change of resistance between stretched and initial state, and ε is the strain.

4 $${\text{GF}} = \frac{\Delta C/C0}{\varepsilon }$$

In this equation, *C*_0_ is the capacitance of strain sensors in an un-stretched state, where Δ*C* is the change of capacitance between stretched and initial state, and ε is the strain.

A linear correlation between the sensor response and the strain deformation is desirable because it makes signal processing simple, thus reducing the complexity and design cost of the signal processing circuits. The changes in the sensing response of the textile strain sensor during the stretching process should also be obvious enough for easy measurement. Moreover, a sensor should exhibit little hysteresis and fast response speed; this type of superiority endows the sensor with the ability to reliably convert strain deformation into electrical signals in real time. Cyclic stability refers to the ability of the resistance to recovery to the initial value after many times loading and unloading, as well as the ability of the sensor to show a similar sensory response in each cycle. High stability should be exhibited during the process of cyclically stretched and recovery. Last but not least, FWSSs should ideally suffer at least 100,000 stretching-releasing cycles for wearable applications considering human joints are frequently in motion [86, 91].

## Conductive Fibers

Fabrication of strain sensors via conductive fibers is a facile and low-cost approach to implement both flexibility and high performance of the devices. Conductive fibers can be developed via spinning, surface modification, and inducing structural transformation, which mainly includes blending active materials into fibers, or modifying existing nonconductive fibers. The spinning technique can massively develop conductive fibers with adjustable conductivity, and controllable diameter and shape by changing spinning parameters such as active material concentration, spinning pinhole and speed. While high conductivity and mechanical performance are not easy to achieve together without further processing. Surface modification is one of the most effective and feasible approaches for the fabrication of conductive fibers, which can be achieved through coating or depositing active elements on fiber surfaces. The conductive elements endow the fiber with good conductivity while poor washability and friction stability. Inducing transformation from nonconductive into a conductive structure is another practical method. A typical example is carbonization, where pristine structures will become carbon-based structures with high-temperature treatment. The conductivity can be simply controlled by changing processing temperature, while the carbonized fibers always lack good mechanical strength.

## Spinning

**Fig. 1:**
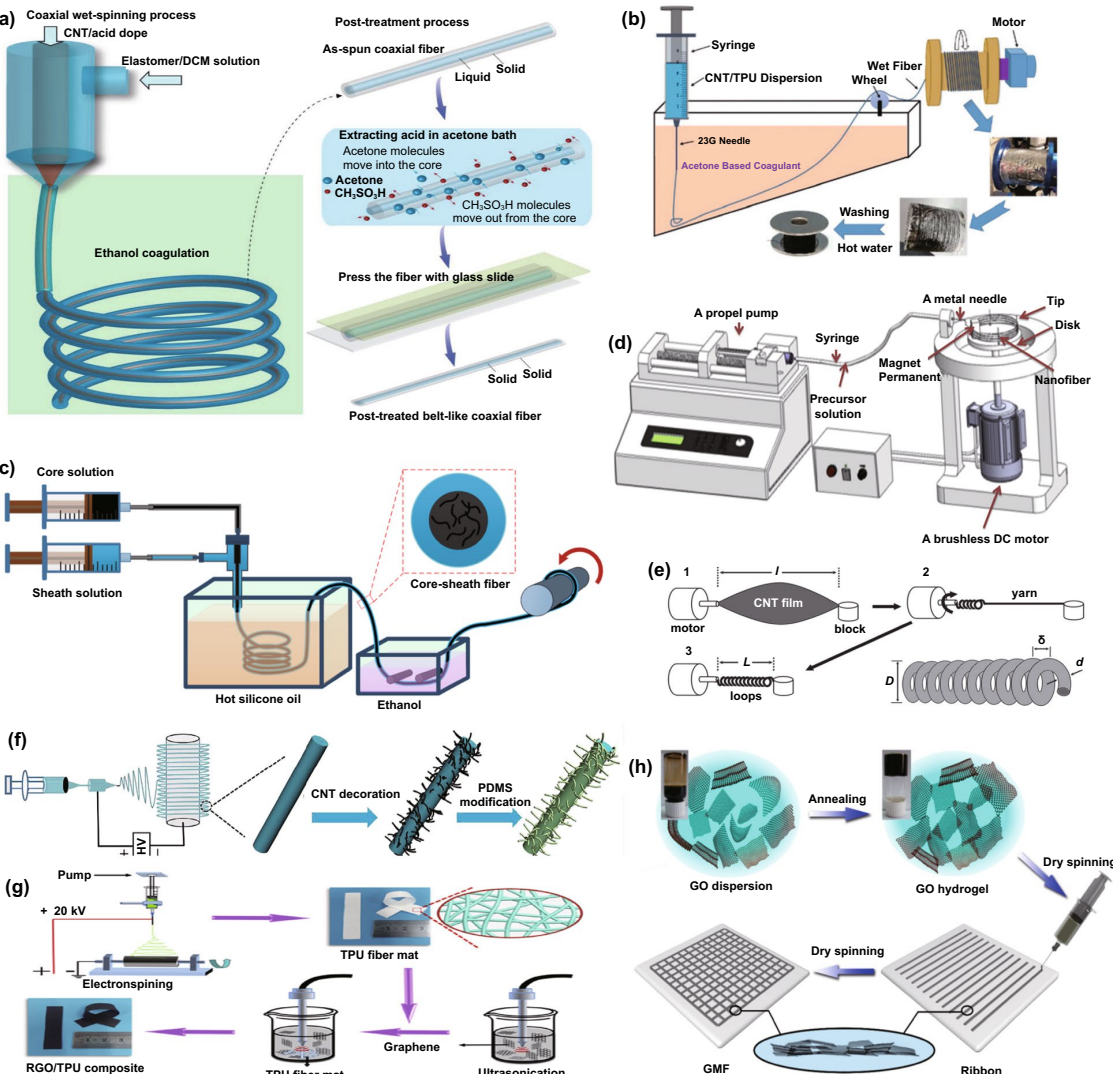
**a** Wet-spinning and post-treatment process for the fabrication of thermoplastic elastomer and CNT composted fibers [96]. Copyright © 2018 Wiley–VCH. **b** The production of thermoplastic polyurethane/CNT fibers for strain sensing via wet-spinning [97]. Copyright © 2019 Royal Society of Chemistry. **c** Wet-spinning process for the fabrication of Ecoflex-30 and CNT composited fibers [98]. Copyright © 2018 American Chemical Society. **d** Schematic diagram of magnetic-mechanical spinning [102]. Copyright © 2017 Elsevier**. e** Schematic diagram of fabrication of spring-like CNT rope from CNT film by spinning technique [103]. Copyright © 2012 Wiley–VCH. **f** Electrospinning TPU fibers modified with CNT and PDMS[104]. Copyright © 2019 Elsevier. **g** Electrospinning TPU fibers modified with graphene for strain sensing [76]. Copyright © 2016 Elsevier. **h** The fabrication of graphene oxide ribbons and mesh fabrics through the dry spinning process [107]. Copyright © 2015 American Chemical Society

Spinning is an efficient way to prepare conductive fibers by directly enabling conductive materials to fibrous structures [92–95]. A versatile method that has been widely utilized for large-scale fabrication of fibers is wet-spinning. In early works, the coaxial wet-spinning approach with chemical reprocessing (Fig. 1a) was demonstrated to prepare belt-like fibers from single-walled CNT for strain sensing [96]. The belt-like fibers possess the width and thickness of 1050 and 200 μm respectively, with good conductivity of 142.6 Ω per 2 cm. After being covered with thermoplastic elastomer sheath, the high-density fragment fibers could detect strain stimulates due to the fragment cracks during the stretching process. Similarly, through wet-spinning, another conductivity-adjustable fiber was developed by mixing thermoplastic polyurethane (TPU) and multi-walled CNT with the weight percentage of 8:1 [97]. The fiber reveals high tensile strength of ~ 28 MPa and a maximum failure strain of 310% (Fig. 1b). By taking advantage of the remarkable mechanical property of TPU and good electro-conductibility of the CNT, the composited fibers were tested to detect tensile strain up to 100% with the high sensitivity of GF ~ 2800. As shown in Fig. 1c, the wet-spinning technique can also be utilized to achieve another performance-controllable core-sheath fiber made with multi-walled CNT and platinum-catalyzed Ecoflex-30 [98]. The dimensions can be well controlled via the regulation of nozzle sizes and flow rates of CNT and Ecoflex-30 inks. By increasing the loaded CNT content, the core–sheath FWSS shows a broader sensing range (> 300% strain) and an outstanding GF of 1378. Besides, other composited fibers such as graphene/polydimethylsiloxane (PDMS) [99], poly(styrene–butadiene–styrene) (SBS)/CNT [100], or TPU/CNT [101] can also be produced via wet-spinning for the assembly of strain sensors. These fibers made by wet spinning mainly include conductive and elastic materials, serving as sensing elements and guarantee mechanical properties, respectively.

In addition to wet spinning, mechanical spinning has also provided a new route to produce strain-detectable fibers. For example, a solution containing polyaniline/polyvinylidene fluoride/γ-Fe_2_O_3_ (PANI/PVDF/γ-Fe_2_O_3_) was combined with conductive fibers through magnetic-mechanical spinning (Fig. 1d), illustrating the advantages of large scale capacity and property controllability [102]. Such fibers with an average diameter of 850 nm made up a twisted rope with high elongation up to the strain of 440% and good conductivity of 6.4 × 10^4^ S cm^−1^. Alternatively, spring-like CNT ropes produced by mechanical spinning with neat and uniform loops over long-distance arrangement were also reported (Fig. 1e) [103], exhibiting an outstanding stretchability and tolerable tensile strain up to 285%. These fiber-based, twisted ropes display great potential in strain detection because of their eminent stretchability and conductivity.

Electrospinning is another commonly utilized spinning technique to produce materials for strain sensing. Generally, the fibers are electro-spun by incorporating conductive fillers such as graphene, CNT, and carbon into the polymer. However, this method deteriorates the electrical properties of the fibers in that the polymer always lacks conductivity. An alternative approach is to produce electrospinning elastic fibers firstly, then modify the fibers with conductive materials. For instance, a previous work prepared an electrospinning TPU fiber, then modified with CNT and PDMS (Fig. 1f) [104]. The conductivity of the fiber-based membranes shows a significant increase trend when enhancing ultrasonication time during the modification process. It can reach 10 S m^−1^ after ultrasonication for 10 min. By taking advantage of the excellent stability of PDMS, such strain sensors can withstand harsh conditions such as acid, moisture, and salt. The concept was also reported in an early work by modifying TPU nanofibers with reduced graphene oxide (rGO) for strain sensing (Fig. 1g) [76]. Several other approaches using modified electrospinning techniques have been demonstrated to yield similarly strain-responsive fibers [105, 106]. Dry spinning [107] and melt spinning [108] were utilized to fabricate strain-detectable fibers in previous studies as well. Early work established a simple dry spinning to prepare graphene mesh fabrics (Fig. 1h) [107]. The mesh consists of several orthogonal GO fibers that can detect strain stimulation due to the high conductivity (703 S cm^−1^) and outstanding tensile strength (582 ± 17 MPa) of the GO fiber.

### Surface Modification

Surface modification on non-conductive fibers, yarns, or fabrics is an effective and practical approach to produce fiber-based, strain-sensible wearable sensors. This approach can be achieved through dip coating, roller coating, spray and rod coatings, printing, as well as surface depositions. The coating technique for strain sensors has the advantages of a wide working range and outstanding sensitivity. However, the long-term performance stability of such devices is not ideal due to the unstable coating layer during strain stimulation; thus, encapsulation is needed in many situations. For example, a bundle of silk fibers was firstly modified by graphite flakes through the rod coating process and then encapsulated with Ecoflex for strain detection [109]. The graphited silk fibers generate considerable breakages and separations during elongation, resulting in resistance increase. Meanwhile, the remarkable elasticity and fatigue durability of Ecoflex enables a stable electrical signal output of up to 3000 cycles with 10% strain. Similar encapsulation approaches were also intensively reported for many inelastic fiber-based strain sensors, such as PDMS encapsulated cotton fibers [110], Ecoflex encapsulated CNT, and silk fibers [90, 111].

**Fig. 2:**
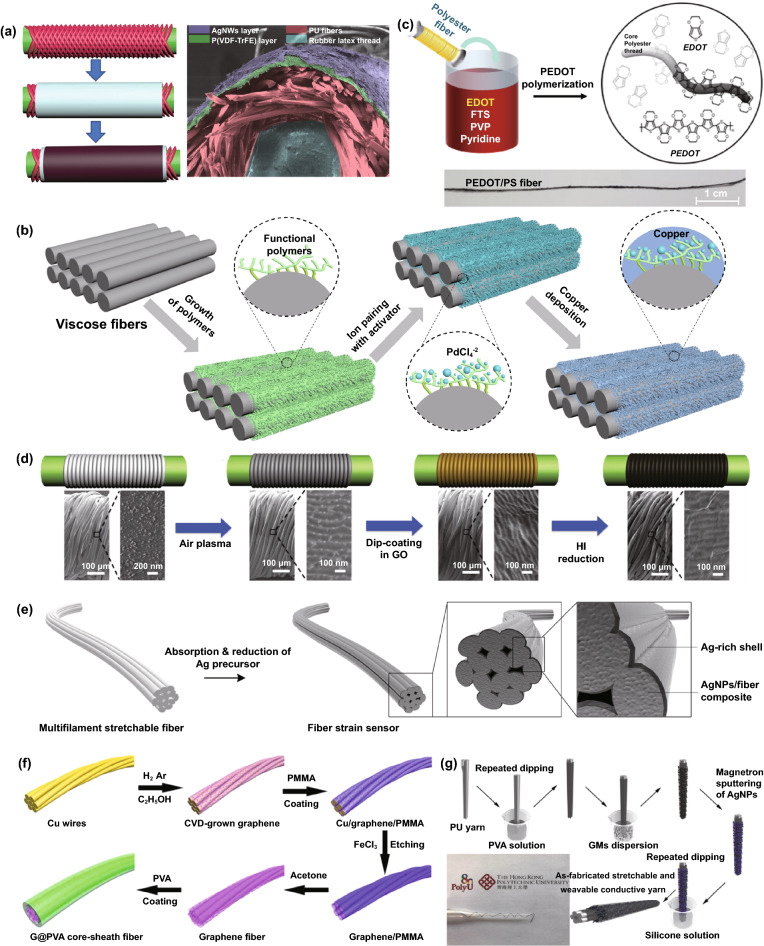
**a** Illustration of P(VDF-TrFE)- and Ag NWs-coated elastic thread for linear strain sensors [113]. Copyright © 2016 Wiley–VCH. **b** Schematic diagram of depositing copper onto viscose fiber surface [6]. Copyright © 2021 Wiley–VCH. **c** In situ polymerization of polyester yarns by PEDOT [119]. Copyright © 2017 American Chemical Society. **d** Fabrication process of a spring-like strain sensor, with the structure of graphene-coated cover layer and PU core. **e** The illustration of Ag modified PU yarn for strain sensor with Ag-rich shell [123]. Copyright © 2018 American Chemical Society. **f** Fabrication of a strain sensor by modifying copper substrate with functional graphene and PVA [124]. Copyright © 2015 American Chemical Society. **g** A 1D coaxial-structured strain sensor consisted of PU core yarn, functional graphene and Ag NPs, and silicon encapsulated layer [125]. Copyright © 2018 Springer Nature

Additionally, surface coating on elastic fibers by conductive elements can also modify the functionalized fiber to detect strain inputs [112]. Previous research developed an Ag nanowires (Ag NWs) dip-coated strain sensor with a core–shell structure. The commercial elastic thread acts as the core substrate, coated by poly(vinylidenefluoride-co-trifluoroethylene) (P(VDF-TrFE)) nanofibers through electrostatic spinning, and the outermost layer is conductive Ag NWs coating (Fig. 2a) [113]. The outstanding stretchability and fatigue durability of elastic thread and P(VDF-TrFE), as well as the high conductivity of Ag, render the composted sensor a wide sensing range more than the strain of 100%, and excellent durability (strain of 5%, frequency of 6 Hz) up to 10,000 cycles. Similarly, another work reported ZnO NWs coated polyurethane (PU) fibers for fabricating strain sensors [114]. The high elastic PU fibers sensors feature a wide working range, up to 150% strain without any degeneration. It is also worthwhile to mention that one previous research exhibited a performance-controllable strain sensor based on Plateau–Rayleigh instability theory, which was manufactured from gold film/CNT coated PMDS fiber with beats [115]. The delicate beats structure of PDMS fiber can redistribute the surface deformation to perceive applied strain, which can be coated by many conductive elements to fabricate strain sensors. Significantly, fiber coating through polymer-assisted metal deposition (PAMD) is a creative approach to developing strain sensors. By coating a variety of metals (e.g., Cu, Ag, Ni, Co, and Au), fiber and many soft substrates could obtain flexibility, stability as well as controllable conductivity, which are significant for the application of strain sensing [116]. In previous studies, Cu and reduced graphene-modified rose petals, and the Ni-modified fibers through PAMD played a function in stretching strain detection [117, 118]. However, such PAMD-functionalized fibers strain sensors showed limited working ranges, needing further enhancement in the future. Alternatively, our previous work reported a strain sensor with high breathability and anti-jamming performance based on a Cu-deposited viscose fiber through PAMD (Fig. 2b). The PAMD was briefly achieved through first-step polymer functionalization, following with ion pairing with palladium moieties as the activator, and finally chemical reaction to obtain dense metal nanoparticles (NPs) onto the substrate surface.

Conductive material-coated yarns are also attractive for developing both linear strain sensors and such yarns-integrated strain sensing devices. Conductive polymers coating is one commonly used approach. In one research, by using in situ polymerization, poly(3,4-ethylenedioxythiophene) (PEDOT)-coated polyester yarns (with the conductivity of 600 Ω/cm after modification) were demonstrated to make up strain sensing elements after being sewed into fabrics (Fig. 2c) [119]. With the advantage of the yarns’ outstanding wearability and assembling flexibility, a sensor-integrated glove was fabricated to rebuild various body motions such as finger bending and other movements. Another similar work reported charged chitosan-modified PU yarn from layer-by-layer dip coating for strain sensing [120]. Nevertheless, without further rubber encapsulation, the sensor shows very limited working range due to unstable attachment of coating layer under large-range elongation. The concept can also be extended to graphene or CNT-modified yarns. More interestingly, the modified yarns that cover elastic yarns form a helix structure, enabling strain sensing ability due to the contact and separation of the modified yarns. Figure 2d shows graphene-coated composites, including the spring-like architecture of functionalized cover layer and PU core [121]. The elastic PU drives the separation of spring-like covering yarns during the deformation process, endowing it with strain sensing ability. Similarly, both CNT-coated cotton yarns and polytetrafluoroethylene (PTFE) thread-covered silicone fiber were demonstrated for strain detection [122]. These spring-like strain sensors present remarkable fatigue resistance by taking advantage of their novel structure, as their coated layer suffers minor damage in repetitious stretching. Elastic yarn-based strain sensors can also be fabricated through metal coating. Figure 2e displays an Ag-coated PU yarn as a strain sensor by modifying a chemical reaction process [123]. The rich-Ag particles formed a dense shell wrapped around the PU fiber, acting as electric response elements. However, even though the device exhibits remarkable sensitivity to tensile strain (GF ~ 9.3 × 10^5^ in the first stretching), the sensitivity cannot be well maintained in the following stretching process. The stability needs to be further enhanced by rubbers or other elastomers encapsulation. Figure 2f illustrates a Cu-based yarn modified by graphene and poly(vinyl alcohol) (PVA) to fabricate strain sensors after attacking the Cu substrate [124]. The sensor with a core–sheath structure presents good electrical conductivity (9.6 × 10^3^ S m^−1^) and mechanical stretchability (590 MPa tensile strength with 16% elongation), paving a facile and straightforward way to develop wearable strain sensors. Figure 2g exhibits a multifunctional strain sensor made with PU yarn, where the substrate is coated with graphene and Ag NPs. The conductive layer is further encapsulated by silicone to ensure its stability [125]. Such sensor processes a wide sensing range (0–50% strain), outstanding sensitivity (GF ~ 500), good durability (2000 cycles), as well as fair linear sensing performance.

**Fig. 3:**
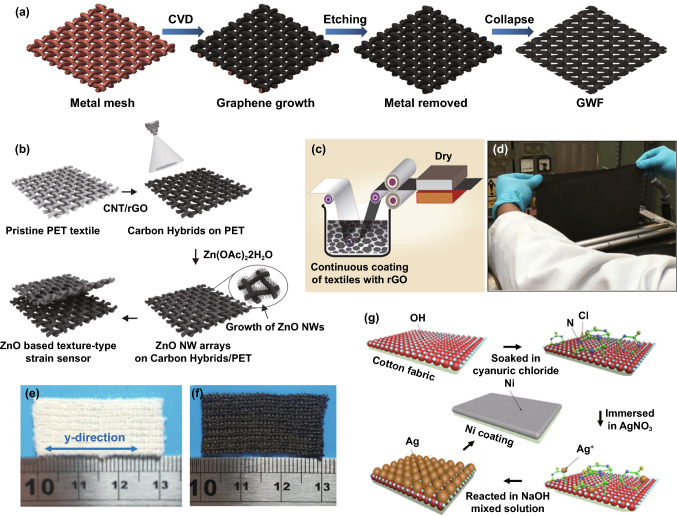
**a** A fabric-like graphene substrate fabricated through CVD growth on a woven copper mesh, and then removing the copper [126]. Copyright © 2012 Springer Nature. **b** A CNT, graphene, and ZnO NWs-coated PET fabric for the detection of bending strain [128]. Copyright © 2016 Wiley–VCH. **c** The process of a graphene-coated fabric by pad dying, and **d** the image of dyed woven fabric [129]. Copyright © 2017 American Chemical Society. **e** The image of a knitting wool fabric and **f** pad-dyed the fabric as a moisture-resilient strain sensor [133]. Copyright © 2020 American Chemical Society. **g** Illustration of step-by-step Ag/Ni coating on woven cotton fabric to fabricate a strain sensor [137]. Copyright © 2018 Springer Nature

Much research in recent years has also focused on functional fabric coatings to develop strain sensors. This type of strain sensor mainly includes the substrates of stretchy fabrics and elastomer-encapsulated inelastic fabrics, of which the substrates are modified with conductive materials such as metals, graphene, CNT, and conductive polymers. A woven copper mesh was modified with graphene through chemical vapor deposition (CVD) growth, and then the copper was removed to get a fabric-like graphene substrate (Fig. 3a), followed by PDMS encapsulation for the fabrication of a strain sensor [126]. This approach was also extended to a graphene-modified woven Ni fabric to produce strain sensors [127]. In Fig. 3b, CNT and reduced graphene are coated as active layers for a polyester fabric to produce a conductive network that will be modified by ZnO NW arrays [128]. The sensor with PDMS protection enables detecting a maximum bending strain of 6.2% with a superior GF∼7.6. In addition to these elastomer-protected fabric sensors, strain sensors can be directly produced by natural stretchy fabrics with conductive material modification. Among them, graphene-coated ones have been reported most extensively. Figure 3c exhibits the pad-dyeing process of reduced graphene functionalized woven fabric. This functionalized fabric (Fig. 3d) presents strain sensing ability due to the separation of coating flakes under tensile strain [129]. However, such woven fabric strain sensors process very minimal sensing range because of the poor stretchability of the woven fabric [130–132]. Alternatively, a graphene pad-dyed knitting wool fabric (Fig. 3e–f) acting as a strain sensor revealed a wider working range of up to 40% tensile strain [133]. By taking advantage of the hydrophobicity of both wool and reduced graphene, the sensor can stably work under complex humidity environments, which is of great significance to human wearable devices. However, the sensor shows poor durability (500 cycles) and obvious hysteresis as well as low recover response speed. This kind of knitting sensors could also be developed through graphene-coated polyester, cotton fabrics [134, 135], as well as CNT-coated cotton fabric [136]. Many fabric strain sensors based on alternative conductive polymers or metal coatings have also been reported, such as Ag/Ni coated woven fabric (Fig. 3g) [137], cellulose nanocrystal/graphene-coated nonwoven fabric [138], and polypyrrole (PPy)-coated woven fabric [139]. Even though these devices can detect strain stimulation, the long-term stability and durability are always not ideal because of the lack of protection, especially under large deformation, which needs to be further optimized.

### Structural Transformation

Some natural and artificial fibers can directly be conductive through structural transformation to produce strain sensors. The electrical conductivity and mechanical properties are well affected by heat temperature. A fundamental study reported that silk fibers suffering sufficient heat with inert atmosphere protection would be conductive, because the silk structure was restructured and transformed into an *sp*^2^-hybridized carbon hexagonal structure with pseudo-graphitic crystalline layers during heat treatment [140]. By taking advantage of this facile carbonization approach, another group first produced ultra-stretchable and highly sensitive strain sensors with carbonized silk fabrics containing Ecoflex encapsulation [90]. Their work investigated the sensor made with different-structure woven silk fabrics, showing the better stability and sensitivity of plain sensors. More importantly, this work has inspired the manufacture of strain sensors through carbonized fibers and fabrics, such as carbonized cotton fabrics, modal fabrics, silk georgette for tensile strain sensors, and carbonized crepe paper for bending strain sensor [141–144]. The carbonization technique has paved a new route for the fabrication of low-cost and high-performance strain sensors, mainly covering the materials of cocoon silk and spider silk, natural and artificial cellulose fibers to date. The conductivity can be simply controlled by changing carbonization temperature. However, this type of material lacks mechanical strength, which needs encapsulation by elastomers in sensor fabrication. More efforts should be focused on the balance between electrical conductivity and mechanical performance in the future.

## Sensing Mechanism

Imparting strain detection ability to the FWSSs generally comes out in various mechanisms. The capacitance and resistance-type strain sensors, which attracted much more attention due to their high sensitivity and facile manufacture, are mainly discussed here with the classification of elastomer-encapsulated and encapsulation-free strain sensors. Their sensing mechanisms also vary, including crack formation, inherent changes of sensing components, disconnection effect, tunneling effect, geometry alteration, etc. Such mechanisms are discussed with representative examples.

### Elastomer-Encapsulated Strain Sensors

**Fig. 4:**
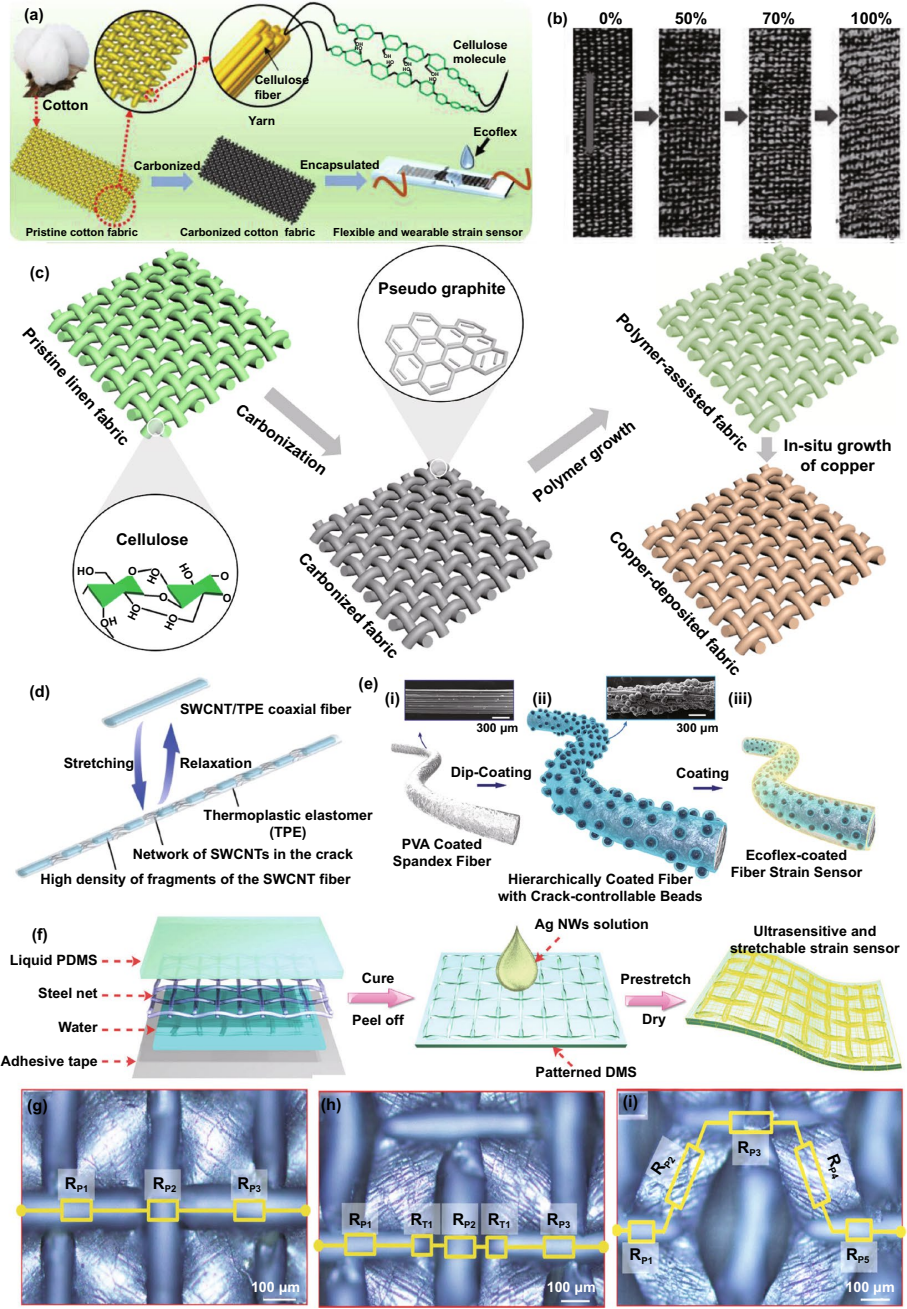
**a** Carbonized cotton fabric with Ecoflex encapsulation for strain sensing, and** b** images of the sensor with diverse applied strain [141]. Copyright © 2017 Wiley–VCH. **c** Fabrication illustration of a strain sensor made with carbonized fabric with PAMD [153]. Copyright © 2021 Elsevier. **d** Illustration of sensing mechanism of yarn-like strain sensors made with thermoplastic elastomer supported CNT [96]. Copyright © 2018 Wiley–VCH. **e** Fabrication illustration of hierarchical strain sensor with spandex fiber substrate, coating of beaded carbon nanomaterials, and Ecoflex encapsulated layer [145]. Copyright © 2019 American Chemical Society. **f** Fabrication illustration of the strain sensor with Ag NWs coating on patterned PDMS, **g–i** schematics of the strain sensor with increasing strain, and corresponding analog circuits [158]. Copyright © 2017 Royal Society of Chemistry

Fiber-based conductive materials supported with elastomers are very popular to fabricate strain sensors, where the fiber and elastomer act as sensing elements and electromechanical insurance components, respectively. The encapsulation thickness is of great importance in determining the flexibility and electromechanical performance of wearable strain sensors. The lower thickness of encapsulation brings about better skin assembling ability while poor stretchability due to possible defects on the encapsulation surface. Increased encapsulation thickness can guarantee good robustness but result in inconsistent compliance between encapsulation and sensing elements, which may deteriorate sensing reliability. Much research in recent years has focused on the relation between strain-caused cracks and the detection capability of strain sensors due to their outstanding sensitivity [145–148]. The unmatched mechanical properties between the fiber-based conductive elements and elastomers lead to functional cracks during strain deformation, further inducing the resistance increase. Figure 4a shows a fabric-based strain sensor made through a carbonization process and Ecoflex encapsulation [141]. Based on the woven structure and hierarchical conductive network, the sensor enables the detection strain of ~ 140% after rupture training. During training, the sensor forms cracks due to the unmatched mechanical properties between carbonized cotton and Ecoflex, exhibiting an increasing resistance with step-up strains (Fig. 4b). This concept was also extensively reported for strain sensing devices in carbonized silk fabric [90], carbonized silk georgette [142], carbonized crepe paper [143], graphene-modified copper mesh [149, 150], graphene-modified cotton fabric with the treatment of ethanol flame [151], CNT mesh [152], and CNT film [2]. Very recently, our group reported a highly sensitive strain sensor based on a carbonized linen fabric with PAMD [153]. Figure 4c exhibits the step-by-step functionalization process: a pristine linen woven fabric is firstly carbonized in a high-temperature environment with the protection of inert gases; a layer of dense copper particles is deposited onto the fiber surface of the carbonized fabric. After the functionalization, the fabric is encapsulated with Ecoflex for the fabrication of a strain sensor. Significantly, unlike most reported strain sensors with single sensing element, the sensor processes two-layer active elements (i.e., carbonized fibers and copper particles), enabling very high sensitivity (GF∼3557.6), stretchability (> 300% strain), the fast response time (225 ms), and good durability (> 6000 cycles). The high sensitivity can be attributed to the significant difference in conductivity and deformability between copper and carbonized fiber. The mechanical and electrical stabilities of these sensing devices are guaranteed by encapsulated rubbers. However, applying excessive strain on these devices with the mechanism of crack formations may destroy the sensing networks, which is still a challenge to be tackled for guaranteeing sensing stability.

In addition to these integrally conductive networks, cracks of functional coatings that form on the surface of substrate fibers also play a vital role in the fabrication of strain sensors. In an early work, a graphene-coated glass fabric with the protection of silicone resin was demonstrated for strain detection, where the silicone resin and graphene were employed to provide flexibility and sensing elements respectively [132]. When stretched, the graphene layer of the sensors experiences the crack formations due to stress concentration, leading to the increase of resistance. After releasing applied stress, the silicone resin drives the contact recovery for reversible strain detection. Interestingly, by taking advantage of the high strength of glass fabric, the sensor maintains structural integrity even sustaining external force over 800 N, which is beneficial to circumvent the damage of excessive tensile deformations. Figure 4d illustrates a yarn-like strain sensor containing thermoplastic elastomer supported CNT produced by a wet-spinning process, of which sensing theory is the crack formation of the CNT with strain stimulation to obtain resistance change [96]. Similarly, Fig. 4e exhibits the fabrication of a hierarchical yarn-like strain sensor, which consists of spandex fiber substrate, coating of beaded carbon nanomaterials, and Ecoflex encapsulated layer [145]. In the above-mentioned research, the sensor’s sensitivity is adjustable by changing the density of microbeads in the carbon coatings. The crack formation in functional coating layers was also studied in graphene-coated stretchable yarns [154], polypyrrole-coated porous PU [155], Ag NWS-coated elastomer with microstructure [156], and carbon black-coated paper [157], which could be utilized in the fabrication of strains sensors.

Encapsulated sensing components with inherent electricity changes are another strain sensors' working mechanism. As shown in Fig. 4f, a strain sensor with an Ag NWs coating is constructed on patterned PDMS, where the microstructure pattern is achieved by pouring liquid PDMS on a fabric-like steel net [158]. The resistance variation of the sensor during deformation is caused by the microstructure change of the conductive Ag NWs network. In further understanding the sensing mechanism, Fig. 4g–i shows surface images and resistance models of the sensor with gradually increased strain. The device resistance increases in the parallel concave lines due to the emergence and increase of tunneling resistance. By utilizing the tensile deformation-induced resistance increase of CNT fibers, they are tightly packed into yarn to detect strain, where the resistance increase is ascribed to the enhancement of bend gap energy of CNT fibers [91, 159]. Besides, ZnO NW-modified carbon fibers, CNT, graphene, and ZnO NW-modified PET fabrics were also demonstrated for strain sensing. The change of piezoelectric potential and band structure of the ZnO layer caused by strain is responsible for the electrical change of the sensors [128, 160].

**Fig. 5:**
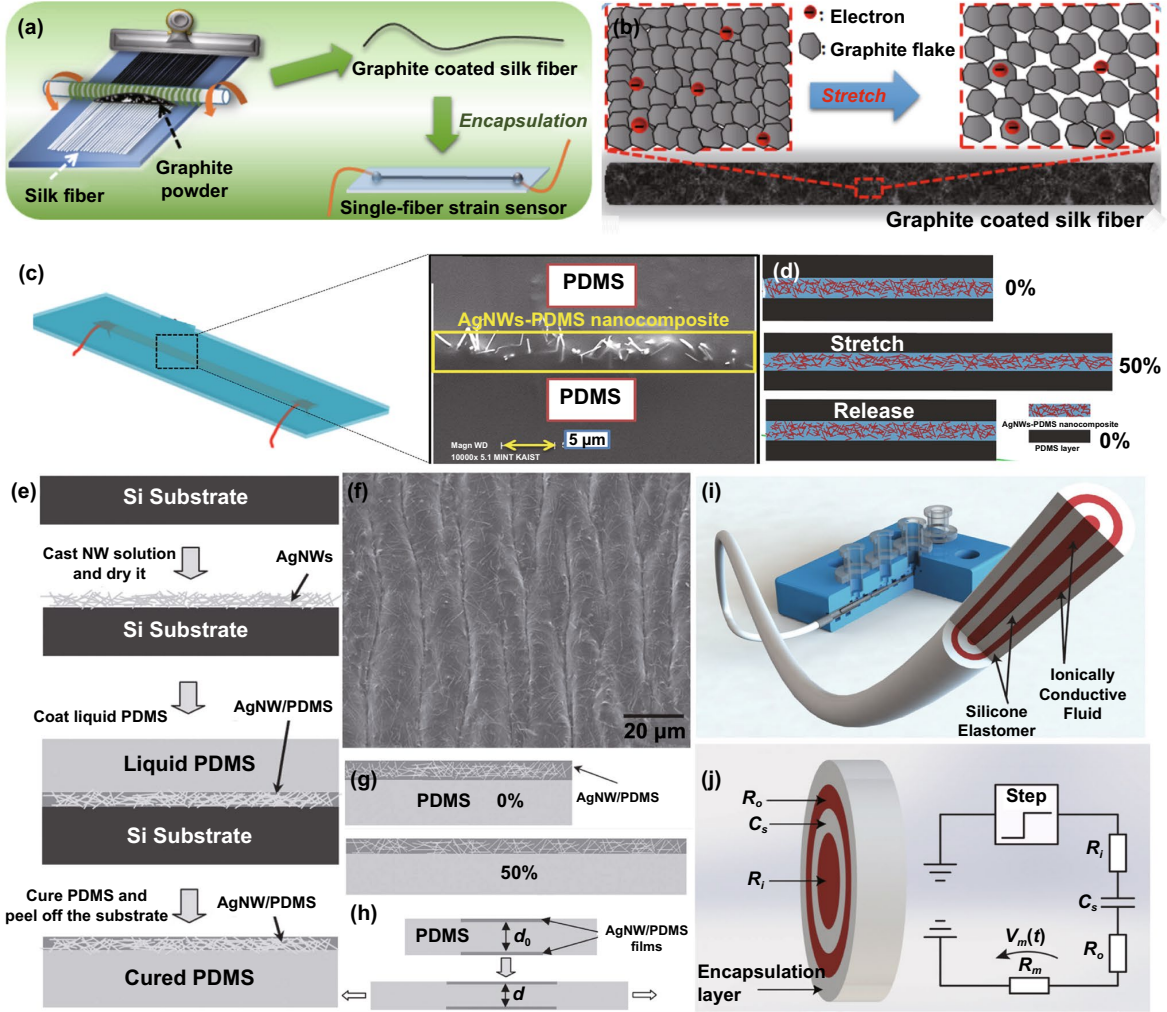
**a** Illustration of strain sensor made with graphite coated silk fiber, and **b** the mechanism diagram of the sensor [109]. Copyright © 2016 American Chemical Society. **c** A sandwich-structure strain sensor with Ag nanowire and PDMS, and **d** corresponding strain-sensing mechanism of the sensor [166]. Copyright © 2014 American Chemical Society. **e** Fabrication illustration of the Ag nanowire/PDMS stretchable conductors, **f** the surface buckling of the conductor after stretching and releasing cycles, **g** the diagrams of the conductor in initial state and 50% strain respectively, **h** fabrication of a capacitance strain sensors with Ag nanowire/PDMS and corresponding sensing mechanism [169]. Copyright © 2012 Wiley–VCH. **i** Illustration of a single-fiber strain sensor by multicore-shell printing process, and **j** corresponding circuit of the capacitance sensor [78]. Copyright © 2015 Wiley-VCH

Inducing disconnection of conductive fibers is also very popular in producing elastomer-encapsulated fiber-based strain sensors. The disconnection will happen on conductive fibers, or the conductive coating layer of a nonconducting fiber. A composite made with CNTs and ethylene-α-octene block copolymer was demonstrated for strain sensing [161]. By utilizing the directional shearing force of melting mixing, the CNTs showed an obvious alignment along the impact direction. The polymer-encapsulated allied CNTs experience two-phase separation with different applied strains: sliding in minor strain and disconnecting in large-grade strain, leading to a diverse sensitivity in the whole sensing range. In Fig. 5a, silk fibers are firstly coated with graphite flakes, and then the silk-graphite composite is encapsulated by Ecoflex to implement the production of low-cost strain sensors [109]. This work also compared the performance difference among several sensors made with graphite coated silk fiber, hair fiber, polypropylene fiber, as well as Spandex fiber. As shown in Fig. 5b, when the graphite-modified silk fiber experiences elongation, the sensor exhibits a resistance climb due to the disconnecting of graphite flakes. Because of the high resilience of Ecoflex, the graphite flakes can recover to the initial contact state after releasing the deformation. The strain sensors with similar disconnection mechanism were also reported in graphene-based, layered percolative film [162], graphene/PDMS mixture [163], and graphene–nanocellulose paper [164]. Figure 5c displays the illustration of producing an Ag NW-PDMS nanocomposite for strain sensing. Similar to the strain sensor made with polystyrene-supported ZnO NWs [165], the sensing mechanism of the sensors can be summarized in two situations: during the elongation, the imposed strain drives the disconnection of conductive NWs to induce higher resistance, while the mutual contact condition will recover to the initial state after releasing the strain (Fig. 5d) [166]. In this work, the performance comparison between two different-structure sensors was also investigated, including simple (Ag NWs embedded on the surface of PDMS) and sandwich-structure (Ag NWs encapsulated with PDMS) nanocomposites. Results show that the sandwich-structured sensor processes a better electrical recovery than that of the simple-structure sample. The reason is that the first-time strain induces the buckling of Ag NWs in sensor with simple structure, and the buckling cannot completely regain after releasing the strain. On the contrary, the phenomenon does not appear to the sandwich-structure sensor in that homogeneous PDMS on the top and bottom of Ag NWs would not result in buckling.

Geometry alteration of stretchable materials based on Poisson’s changes plays a critical role in regulating the strain detecting abilities of fiber-based, resistive and capacitive strain sensors [86, 87, 162, 167]. Elongation-induced electrical changes can be attributed to the increasing length and decreasing sectional area, while the electrical resistivity remains stable for strain sensors with the mechanism of geometry alteration. For example, elastic hollow fibers composed of a triblock copolymer, poly[styrene *b*-(ethylene- *co*-butylene)-*b*-styrene] resin, were fabricated into strain sensing devices. A liquid metal alloy (i.e., eutectic gallium indium) was subsequently injected into the cavity for better conductivity [168]. Similarly, another work reported the fabrication of strain sensors by injecting low toxicity liquid metal into hollow PDMS fibers [74]. These sensors enable to detect strain variation by the strain-induced sectional area changes and fiber length changes, of which mechanism can be classified into geometry alteration. For capacitive sensors, the capacitance increases during stretching because of the additional length and decreasing thickness of the dielectric components. Figure 5e shows the fabrication process of stretchable Ag NWs conductor by combining with PDMS. The conductor experiences a surface buckling after cyclic stretching and releasing (Fig. 5f), while the buckling will gradually disappear in high-grade elongation (Fig. 5g) [169]. By utilizing this patterning technology, a capacitance strain sensor containing two Ag NW conductors in the top and bottom surfaces of PDMS was produced (Fig. 5h). In another work, a piece of cotton fabric was firstly modified by PAMD, and then this metal-deposited fabric was coated by PDMS to protect the metal layers [170]. A capacitance sensor made with these two face-to-face fabrics was studied to explore the relationship between two fabrics’ capacitance and spatial location differences. Besides, a fancifully constructed single fiber in Fig. 5i by multicore–shell printing approach was produced for strain sensing. The sensor consists of alternating layers of ionically conductive fluid and a silicone elastomer, serving as the conductor and dielectric/encapsulant, respectively [78]. Figure 5j exhibits the schematic cross section of the sensor and corresponding circuit, whose sensing mechanism is also attributed to the geometry alteration of the four concentric components during the stretching process.

### Encapsulation-Free Strain Sensors

Fiber-based strain sensors without the protection of elastic encapsulation layer are significant for truly wearable applications, as they allow metabolite excretion from the skin surface to the environment due to their porous structure [57, 133, 171, 172]. These sensors can be achieved by making conductive fiber into yarns and fabrics or modifying existing yarns or fabrics with active components. In the cases of the fabrication of strain sensing fabric, conventional textile forming technologies such as knitting, weaving, braiding, sewing, and nonwoven approaches are very popular due to their pattern flexibility, low consumption, and large-scale production. The fabrication and performance of some reported strain sensors by textile forming technology are summarized in Table 1.

**Table 1:** Summary of reported fiber-based strain sensors made with textile forming technology

Substrate	Types	Active materials	Strain range	GF	Cycling stability	Refs
Pu fiber	Resistive	Ag NPs	200%	35 (0–100%), 659 (150–200%)	10,000 (10%)	[123]
Pu fiber	Resistive	ZnO NWs	> 150%	15.2 (0–10%), 4.1 (10–150%)	> 10,000 (8.7–23.2%)	[114]
Polyolefin yarn	Resistive	Ag NWs	64%	13,920 (64%)	4500 (10%)	[173]
Elastic yarn	Resistive	Ni/Cu NPs	1100%	–	5000 (50%)	[174]
PET/elastic woven fabric	Resistive	PPy	105%	51.2 (0–40%), 27.6 (40%– 105%)	400 (10%)	[175]
PDMS woven fabric	Resistive	rGO	30%	70 (10%), 282 (20%)	–	[176]
Silk woven fabric	Resistive	carbonized silk	> 500%	9.6 (0–250%), 37.5 (250–500%)	6000 (100%)	[90]
Cotton woven fabric	Capacitive	Ni NPs	–	–	3600 with bending angle of 90°	[67]
Silk knitted fabric	Resistive	rGO	10%	124.5 (10%)	1000 (2%)	[177]
Spandex/polyamide knitted fabric	Resistive	Carbon black	30%	62.9 (0–30%)	5000 (10%)	[134]
Pu knitted fabric	Resistive	Ag NWs	140%	10.3 (0–60%), 6.3 (60–140%)	2500 (10%)	[178]
Nylon/Pu knitted fabric	Resistive	rGO	30%	18.5 (0–10%), 12.1 (10–18%)	120 (3%)	[135]
Wool knitted fabric	Resistive	graphene	40%		500 (20%)	[133]
TPU nonwoven	Resistive	rGO	98%	180 (15%), 23,600 (98%)	1000 (10%)	[138]
PVdF-HFP nonwoven fabric	Capacitive	SWCNT	0.3%	134 (0.3%)	1000 (0.1%)	[179]
TPU mat	Resistive	rGO	100%	11 (10%), 79 (100%)	6000 (50%)	[76]
Embroidery	Resistive	Ag	100%	0.1 (0–49%), 42.9 (50–57%)	2500 (40%)	[180]
Embroidery	Resistive	Ag	21%	–	4000 (10%)	[181]
Embroidery	Resistive	graphene	–	0.13 (0–8.85%) 0.37 (8.85–12.5%) 0.94 (12.5–13.64%)	2800 (40%)	[182]
Embroidery	Resistive	Cu	200%	49.5 (0–100%) 23.7 (100–150%) 6.9 (150–200%)	3000 (50%)	[6]

**Fig. 6:**
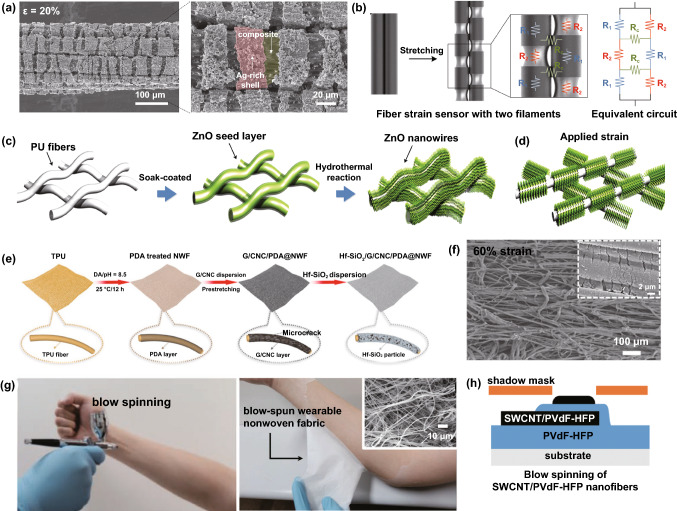
**a** A yarn-like strain sensor fabricated by modifying PU yarn with AgNPs, and **b** the corresponding mechanism of the sensor [123]. Copyright © 2018 American Chemical Society. **c** The fabrication of a strain sensor by modifying PU fiber with ZnO NWs through the layer-by-layer coating, and **d** the crack formation on the sensor to show the sensing mechanism [114]. Copyright © 2016 Wiley-VCH. **e** The fabrication of a tunable wearable strain sensor with self-cleaning capability by layer-by-layer multifunctional coating, and **f** the crack formation of the coating layer to show the sensing mechanism [138]. Copyright © 2019 Elsevier. **g** The fabrication of nonwoven fabrics through blow spinning, and **h** the fabrication of a capacitive strain sensor by vertically stacking the PVdF-HFP dielectric fabric and the CNT/PVdF-HFP conductive fabric [179]. Copyright © 2019 Wiley-VCH

Elastic fibers with the modification of conductive components are facile candidates to produce strain sensors. A yarn-like strain sensor by incorporating Ag NPs into a PU yarn was fabricated, and the surface coating would experience the formation of cracks when the strain was applied (Fig. 6a) [123]. To further understand the mechanism, a resistance model of two filaments of the sensor in Fig. 6b shows the three types of resistance. *R*_1_ represents the resistance of the Ag-rich shell grabs the inner composite, *R*_2_ refers to the resistance of the exposed conductive composite, and *R*_C_ is the contact resistance between the Ag-rich shell regions in neighboring filaments. As Ag separates under strain, *R*_2_ becomes infinite, and the system's resistance increases along with the imposed strain. Similarly, a strain sensor was also fabricated by modifying PU fiber with ZnO NWs through the layer-by-layer coating (Fig. 6c), and the sensing mechanism was attributed to the crack formation on the ZnO layer as well (Fig. 6d) [114]. In addition to the surface modification on stretchable yarns, a yarn-shaped strain sensor could also be made by mixing Ag NWs and polyolefin elastomer nanofibers into a single yarn, with the advantages of low-cost and large-scale production [173]. The sensing mechanism of the sensor can be classified into the disconnection effect. The functional fibers (i.e., Ag NWs) undergo pathway breakage when the composite yarn is elongated, and the pathway will recover to its initial state after strain is released. Figure 6e exhibits the processing scheme of a PU nonwoven fabric. The TPU fabric is firstly treated by polydopamine solution and then coated by conductive cellulose nanocrystal and graphene aqueous dispersion. In the following process, the stretching takes place to form microcracks on the surface, and the processed fabric will finally be transferred into the solution of hydrophobic fumed silica [138]. By adjusting the pre-elongation degree and applying hydrophobic fumed silica, multi-functionalized elastic fabric-based strain sensors exhibit controllable sensitivity and water resistance. The functionalized cracks on the surface of the elastic PU fiber endow the fabric with strain sensing ability (Fig. 6f), and the hydrophobic coating layer renders it self-cleaning capability. Also, a stretchy poly(styrene-block-butadiene-block-styrene) (SBS) nonwoven fabric with the surface modification of Ag NPs was demonstrated for strain detection, of which sensing mechanism could be attributed to the crack formation of the functional layer as well [183]. Flexible strain sensors can also be made by modifying graphene on inelastic fibers in addition to elastic fiber-based nonwoven fabrics. A nonwoven fabric made with polyester and a small amount of viscose fiber was fabricated as a strain sensor, experiencing GO coating and reduction processes [184]. The strain-induced contacting of the functional fiber increases the sensor resistance, and it can recover to the initial state after releasing strain. Even though the fabrication with the advantages of low-cost and large-scale capability, the sensor only processes a very limited sensing range. Figure 6g illustrates a very novel approach of producing two blow-spun nonwoven fabrics made with pure poly(vinylidenefluoride-co-hexafluoropropylene) (PVDF-HFP) and CNT [179]. A wearable capacitive strain sensor was developed by vertically stacking the PVDF-HFP dielectric fabric and the CNT/PVDF-HFP conductive fabric (Fig. 6h). The limitation of narrow sensing range emerges among the sensors, although their fabrications have the advantages of low-cost and large-scale capability.

**Fig. 7:**
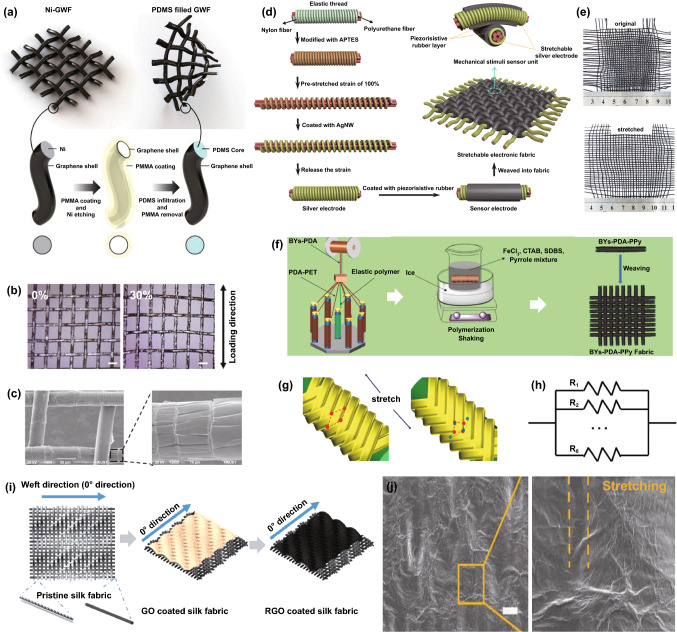
**a** A woven-structure strain sensor by depositing graphene on a nickel mesh and replacing the nickel by PDMS, **b** the surface morphologies of the sensor under 0 and 30% strain respectively, and **c** the crack formation with applied strain to illustrate the sensing mechanism [176]. Copyright © 2019 American Chemical Society. **d** Illustration of a woven strain sensor through weaving Ag NWs and piezoresistive rubber-coated yarns into a fabric, and **e** the images of the fabric sensors in the original and stretching state [185]. Copyright © 2016 Wiley–VCH. **f** Fabrication of a strain sensor by weaving conductive polymer-modified yarn into a plain fabric, **g** the illustration of the yarn without and with stretching, and **h** the resistance model of the sensor showing the sensing mechanism [175]. Copyright © 2019 American Chemical Society. **i** The process of coating graphene on the surface on a woven silk fabric as a strain sensor, and **j** crack formation on the graphene layer to show the mechanism of the sensor [177]. Copyright © 2020 Wiley–VCH

Woven is a very proven technique to integrate orthogonal warp and weft yarns into fabrics, which is also attractive to produce breathable strain sensors. The woven-structure sensors can be developed through modifying existing fabrics with conductive elements or weaving conductive yarns into fabrics. Figure 7a shows a spiderweb-inspired strain sensor with a woven plain structure, whose both warp and weft yarns are made with PDMS core and graphene shell [176]. Three main processes produce this spiderweb-like fabric sensor: graphene is firstly deposited on the surface of a nickel mesh, and then a layer of polymer is coated, following the etching of nickel component; finally, the nickel interspace is filled with PDMS solution. The parallel yarns in the fabric strain sensor experience separation from each other during elongation (Fig. 7b), and strain-induced cracks of the graphene layer in Fig. 7c explain its sensing mechanism. Figure 7d shows the fabrication of another woven strain sensor, made with intertwined and functionalized yarns [185]. To produce the functionalized yarn, the pristine core yarn with inner PU fibers and helically-wounded nylon fiber is firstly modified by Ag NWs in a stretched state, after which the strain will be released for coating piezoresistive rubber on surface. The functionalized yarns are weaved into a fabric (Fig. 7e) for strain sensing, where the conductive yarn separation serves as the sensor mechanism. Very similarly, a woven strain sensor with the same structure was reported in another work. The PET yarn was immersed in dopamine solution, and then the processed PETs were braided on an elastic core yarn surface, followed by the reaction with conductive polymers (Fig. 7f) [175]. By integrating the as-fabricated yarn, the woven fabric achieved strain sensing ability. Figure 7g illustrates the sensing mechanism by showing that the increasing elongation causes the fibers to wind at a greater angle and distance, resulting in a lower ability to conduct electricity. The several conductive PET fibers acting as separate resistances with parallel connection (Fig. 7h) can further explain the resistance change, as the increase in value of each paralleled resistance will contribute to the augment of the overall resistance. Coating conductive elements on pre-existing fabrics can also produce the woven-structure strain sensor. Both elastic and inelastic yarns could be chosen as fabric raw materials [186, 187]. Figure 7i displays the processes of coating GO on pristine woven silk fabric and reducing the GO with a hot press to fabricate strain sensors [177]. Although the silk yarn has little stretchability, the woven structure endows the sensor with strain sensing ability in a limit range of up to 10% strain. Apart from that, this work also investigated the sensitivity of the sensors with different stretching angles. The results show that 0°-direction (along with the twisted yarns) sensor owns better sensitivity due to the more microcracks on the graphene layer than other sensors (Fig. 7j).

**Fig. 8:**
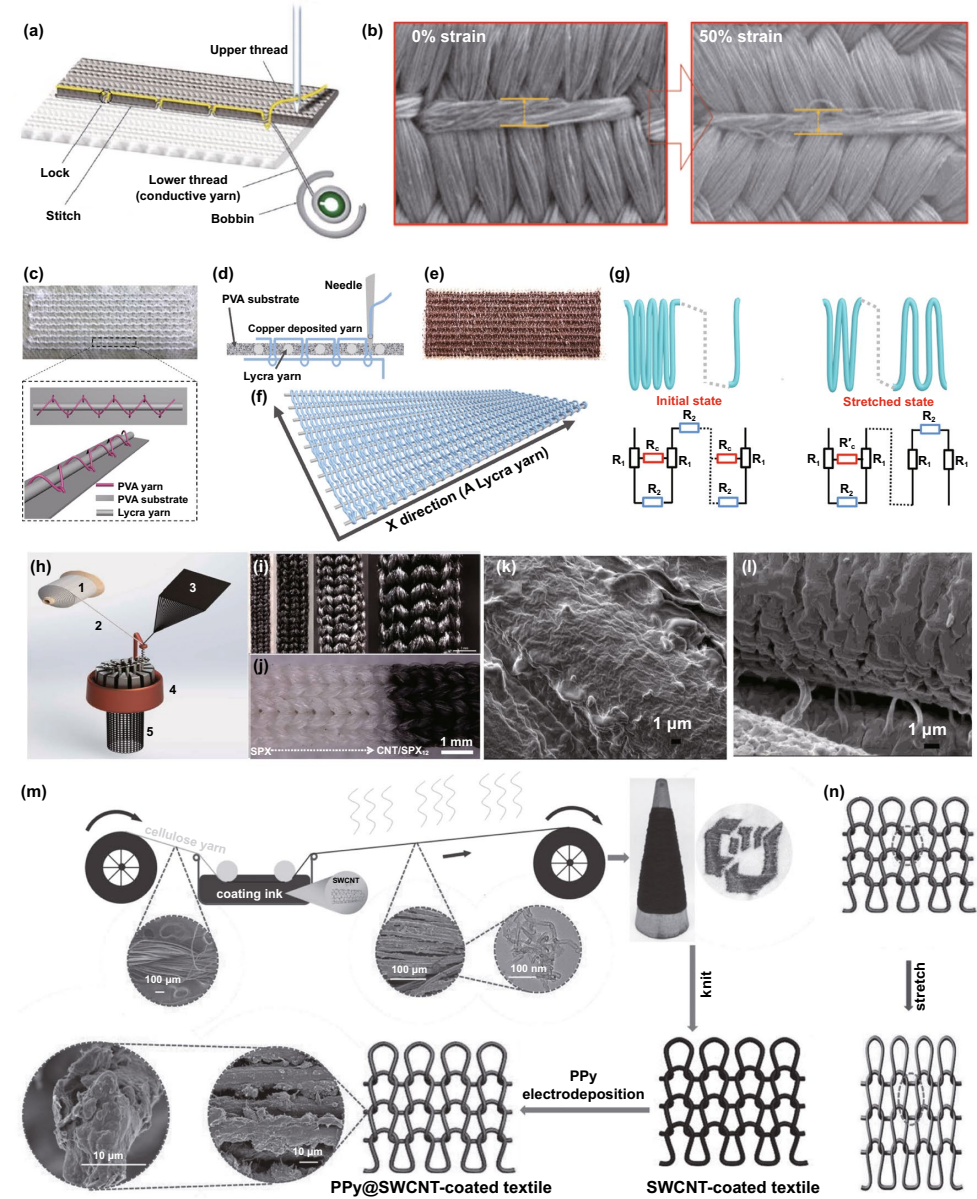
**a** Illustration of locking conductive yarns over a textile substrate to fabricate strain sensors, and **b** the surface morphologies of the strain sensor under 0 and 50% strain respectively [180]. Copyright © 2019 Wiley–VCH. **c** A rectangular Lycra track fixed onto PVAT substrate by PVAT yarn, and **d** the diagram of embroidering copper deposited yarn in the Lycra track via lock-stitch embroidery to fabric strain sensors. **e** The photo of the embroidery pattern with copper deposited yarn and the Lycra track. **f** The embroidering sensor after dissolving PVAT substrate. **g** The sensing networks and resistance models of the embroidery sensor under initial and stretched states respectively [6]. Copyright © 2021 Wiley–VCH. **h** Illustration of fabricating a knitted Spandex/CNT strain sensor through a circular knitting machine, **i, j** different-structure Spandex/CNT sensors showing the pattern flexibility of the technique, **k** the surface CNT on the pristine Spandex fibers, and **l** the CNT cracks on the stretched Spandex fibers to showing the sensing mechanism [188]. Copyright © 2016 American Chemical Society. **m** The process of producing a knitting strain sensor through coating CNT and PPy on cellulosic yarns, and **n** the schematic diagrams of the sensor without and with elongation, showing the mechanism of the sensor can be attributed to geometry effects [189]. Copyright © 2016 Wiley–VCH

By making use of pattern flexibility of sewing, embroidering, and knitting, strain sensors can be achieved with desired performance. Figure 8a exhibits the fabrication of a strain sensor by sewing conductive yarns over a stretchable textile substrate [180]. This work investigated the performance variation of the sensors with different stretching degrees of substrates. Results show that the sensor with a lower stretched substrate owns a more significant and earlier strain response. The strain response can be ascribed to the disconnection and connection of the conductive lockstitches with strain applying and releasing respectively (Fig. 8b). More importantly, by adjusting the pre-strain of the substrate, the work firstly developed a strain sensor that could program the starting point of strain detection. We recently reported a high-performance strain sensor fabricated through a novel embroidery technique with an insensitive response to both bending and pressuring inputs [6]. An elastic Lycra yarn was firstly fixed onto a water-soluble polyvinyl acetate (PVAT) substrate by water-soluble PVAT yarn (Fig. 8c), and a copper-deposited conductive yarn was then stitched onto the Lycra yarn and PVAT substrate (Fig. 8d) to obtain the primary sample (Fig. 8e). After immersing the primary sample into water for removing PVAT yarn and substrate, we finally fabricated a strain sensor constructed with elastic Lycra yarn in *X* direction and copper-deposited conductive yarn in *Y* direction (Fig. 8f). Figure 8g presents the geometry diagrams and electrical models of the sensor in initial and stretched states respectively. It illustrates that parallel circuits gradually transform into series circuits with increasing deformation, thus leading to resistance increase. The significance of this work is that it emphasizes sensor breathability for wearing comfort and anti-jamming for practical applications.

Developing strain sensors with knitting structures is also one of the most popular approaches, as knitting fabrics are naturally stretchable due to their unique interconnecting loops. Figure 8h shows the knitting process of Spandex/CNT fabric through a circular knitting machine. In this process, both fabric structure and density can be well controlled by changing the forms of CNT and Spandex (Fig. 8i–j) [188]. The formation of microcracks on the sensor surface can be observed from Fig. 8k-l, which correspond to the unstretched fabric and stretched fabric respectively. Apart from that, experiencing a strain-induced separation of the interconnected conductive yarns will also increase the sensor’s resistance. Consequently, the strain sensing mechanism of the sensor can be attributed to both crack formation and geometric effect. Figure 8m exhibits another knitting strain sensor made with CNT and PPy coating on cellulose yarns [189]. As shown in Fig. 8n, the gaps between the conductive yarns become narrower during elongation, leading to more conductivity paths and driving lower overall resistance. Such knitting strain sensors follow a geometry change with stretch stimulation [95, 133, 134]. However, some knitting strain sensors indicated increasing resistance with applied strain [190–192]. How knitting parameters such as structures, density, and yarn diameter determining the sensor performance should be further investigated in the future.

## Applications

Fiber-based strain sensors have great application potential in a variety of wearable occasions. By converting strain-induced deformation into visible electrical signals, the devices can monitor body motions and vital signs with the advantages of accuracy, portability, and shortcut. Moreover, strain sensors integrated sensing array enables to detect resolved strain. The sensing array could be utilized for the applications of human–machine interaction and future entertainment.

### Body Monitoring

#### Motion Sensing

**Fig. 9:**
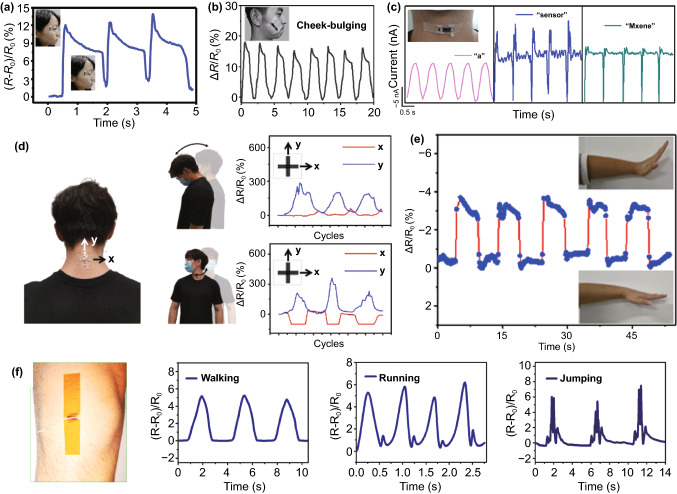
**a** The monitoring of the blinking by attaching a strain sensor to the corner of one eye [90]. Copyright © 2016 Wiley–VCH. **b** Detection of the signals generated by cheek bulking [141]. Copyright © 2017 Wiley–VCH. **c** Real-time recording and recognition of speaking different words [193]. Copyright © 2019 Wiley–VCH. **d** Detection of neck movements through a multidirectional strain sensor [195]. Copyright © 2019 Wiley–VCH. **e** Monitoring the motion of wrist bending by attaching the miniaturized strain sensor to the wrist [199]. Copyright © 2020 American Chemical Society. **f** The detections of walking, running, and jumping by mounting a strain sensor on the knee surface [201]. Copyright © 2018 American Chemical Society

By attaching skin-mountable, fiber-based wearable strain sensors to different body parts, body movements ranging from small to large deformation can be monitored in real-time. A previous achievement introduced a strain sensor made with Ecoflex-encapsulated carbonized silk fabric, which could detect blinking (Fig. 9a) [90]. The sensor was attached at the corner of one eye to sense tiny muscle movements of blinking. The outcomes exhibit that resistance change is highly consistent with corresponding motions. Figure 9b displays the real-time detection of another subtle facial movement by attaching a rubber-supported carbonized cotton sensor [141]. The resistance firstly increases with the cheek bulging and then gradually drops to the initial level, which is promptly in agreement with the facial motions. The wearable strain sensors can also record the epidermal vibration of the throat generated by speaking, water drinking as well as swallowing. Figure 9c shows a strain sensor attached to the throat, which can record the electrical signals resulting from speaking different words [193]. By clarifying different electrical outputs, the sensor can be utilized to differentiate monosyllable alphabet (e.g., a) or complex polysyllabic words such as “sensor” and “MXene,” of which signals present tiny peaks consistent with corresponding syllables. This real-time and precise detection reveals application potential in phonation reconstruction and singing training. Besides, placing a strain sensor on the throat could monitor the acts of lowering or raising heads, drinking, and swallowing [133, 194]. However, affected by the differences of human throats and sensing locations on the throat, signals collected by strain sensors present a reverse trend, which should be investigated in the future [90, 133, 134, 141].

For larger deformation detection, many significant breakthroughs have also been made. A multidirectional strain sensor, which contained multiple degrees of freedom, was attached to a human neck to monitor the motion of the neck (Fig. 9d) [195]. With repetitive ventral neck actions, there was a reduplicative increasing and decreasing resistance change in the *y*-direction, while the value maintained stable in the *x*-direction. When the motion became into rotational movements, different signals were observed. The strain sensors could identify the degree of bending fingers, wrists, and arms by altering the electrical signal [196–198]. Figure 9e exhibits an ultraminiaturized strain sensor acting as an e-skin to detect the wrist bend and release [199]. With the wrist bending up and back down, the relative resistance firstly increases and then recovers to the initial state, of which signals are in accordance with corresponding motion input throughout the cycles. Taking advantage of the ultraminiaturized structure, dense arrays made with multiple sensors are expected to be applied as the mapping of strain distribution. For instance, the angle of elbow bending was detected by wearing a capacitive-based strain sensor on the elbow [200]. During the process of step-by-step flexion and extension, corresponding relative capacitance change was simultaneously recorded. This real-time elbow angle measurement system can be utilized in body posture capture, motion state monitoring, and athletic training. In Fig. 9f, a strain sensor is worn on the knee surface to detect walking, running, and jumping [201]. The speed and frequency of the motions can be easily detected by collecting the data of relative resistance changes, which has a highly repeatable manner with consistent signal-to-noise ratios for the cycles of each motion. In addition to these abovementioned examples, various body motions such as body swaying, bicycling and squatting were also successfully monitored by wearable strain sensors in real-time [158, 202, 203]. By applying strain sensors with suitable working ranges, almost all body movements can be monitored. It is worth mentioning that the data can be collected by wireless devices, even if the strain sensor is implanted in the body [3, 128]. Fiber-based strain sensor with good biocompatibility is promising in biomedical and implantable applications to monitor connective tissue strain [204]. It can perform the measurements of the knee ligament and achilles tendon deformation of a pig, showing potential in the in vivo detection of body motion in the future.

#### Health Indicators

**Fig. 10:**
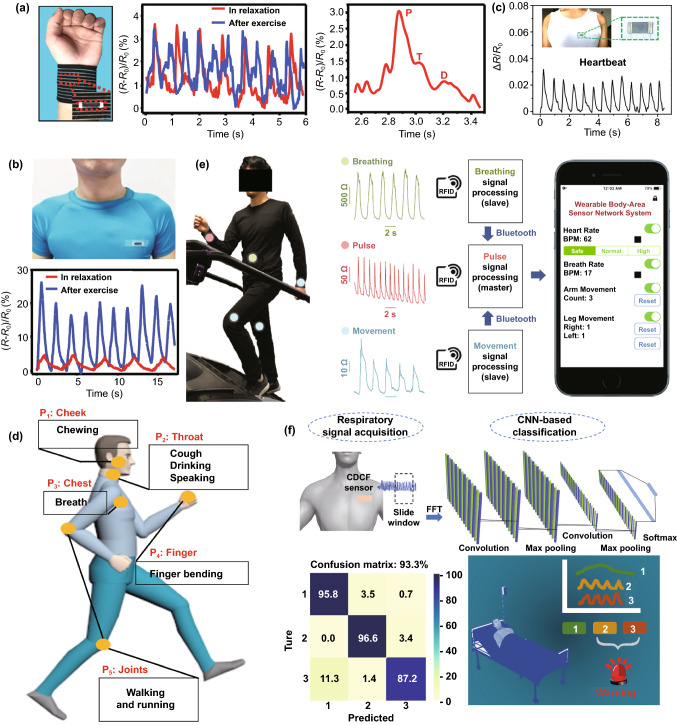
**a** The detection of the wrist pulse by assembling a carbonized silk fabric-based strain sensor on the wrist surface, and **b** real-time respiration monitoring by attaching the sensor to the location of chest surface [90]. Copyright © 2016 Wiley–VCH. **c** The signals of heartbeat collected by a PDMS-supported conductive polymer [205]. Copyright © 2018 American Chemical Society. **d** An overview of the locations of body sensing networks based on embroidery strain sensors [6]. Copyright © 2021 Wiley–VCH. **e** The photograph of body sensing networks in monitoring breathing, pulse, and arm movement [206]. Copyright © 2019 Springer Nature. **f** A respiration monitoring and emergency warning system based on a strain sensor and a deep learning network [153]. Copyright © 2021 Elsevier

Personal and public healthcare management plays an essential and indispensable role in social governance, epidemiology, and disease control. Wearable strain sensors may contribute to the large-scale classifier of individuals and the public accurately and unobtrusively. High-detection-limit and fiber-based strain sensors are capable of sensing vital sign signals such as heartbeat, pulse, breathing with tiny deformation, which makes them become suitable candidates as health indicators to monitor human health timely. In Fig. 10a, a design for detecting the wrist pulses is shown by mounting a strain sensor made with Ecoflex-encapsulated carbonized silk fabric on the wrist surface [90]. Two-state wrist pulses under relaxed and exercised conditions can be effectively characterized by capturing changes in the relative resistance of the sensor. The repeatable and regular pulse shapes reflect the frequency of the wrist pulse in relaxation, reaching 70 beats min^−1^. In comparison, the value ascends to 110 beats min^−1^ after exercise, because of the exercise-induced enhancement of metabolism. The close-up of one pulse waveform under relaxation conditions presents the percussion wave (P-wave), tidal wave (T-wave), and diastolic wave (D-wave), which exhibits the remarkable sensitivity of the sensor. The highly sensitive strain sensor is also capable of perceiving human respiration by assembling it on the chest. Relative resistance change in Fig. 10b reveals that relative resistance change can reflect the respiration under relaxation and exercise. The waveform for the respiration under relaxation exhibits a lower peak and frequency, while during exercise, it has a higher peak and frequency, consistent with the two-state respiration. Heartbeat, as an important vital physiological signal, closely relates to human health conditions. With the assistance of fiber-based wearable strain sensors, some diseases such as sudden infant death syndrome can be diagnosed effectively by detecting heartbeat. An application of conductive polymer-supported strain sensors is shown in Fig. 10c [205]. By integrating the sensor on a tight and wearing it on the chest, the signals generated by the heartbeat show outstanding accuracy and remarkable repeatability throughout. It also manifests that the heartbeat reaches a frequency of ~ 1.5 beats s^−1^.

In addition to the abovementioned health condition detection in a single body position, strain sensors can be integrated into body sensing networks (BSNs) to monitor full-range vital signs. Our previous work showed a BSN based on high breathable and stretchable strain sensors (Fig. 10d) [6]. Because of its high sensitivity in the wide sensing range, the strain sensor in this system can detect various physical movements, from tiny breath signals to large motions from knees and fingers. By attaching sensors to different body positions, these all fiber-based structure sensors provided the information of full-range body motions in real-time with high comfort and accuracy. A reliable BSN system utilizing Radiofrequency Identification (RFID) technique was reported (Fig. 10e) [206]. The five on-skin stretchable passive tags with thickness-gradient CNT network made contact with clothing via on-textile circuits. Significantly, the RFID technique helped researchers to achieve an all-flexible system. The absence of rigid substrates relieves integration density, providing better durability and enhancing the wearing comfort of this network. By collecting and integrating three different vital signals (breathing, pulse and joints activity) and sending them to the smartphone wirelessly, this strain sensing network illustrates its promising future in personal health care and exercise monitoring.

Apart from RFID, Near Field Communication (NFC) is also a reliable communication technology for building wearable wireless sensing networks. By integrating conductive thread and relays on clothing with a programmable pattern, Lin et al. fabricated a fabric-based sensing network, where clothes act as multiple relays system to connect with strain sensor nodes [207]. Researchers succeeded in monitoring various motions by changing the location of layers and sensors. For instance, the sensors and NFC labels in the cervical, thoracic, and lumbar sections provided instantaneous posture monitoring for different bending movements. Sensors were also integrated into running pants for monitoring knee movement. A clear stride time decrease from 0.98 to 0.65 s was collected when the tester increased his running speed from 2.8 to 9 km h^−1^, closely corresponding to the interval change from data of gyroscopes. Temperature sensors were also added into the network; these systematic data may provide an overall result for athletic activity or medical testing in a convenient method. Different from the RFID route, the usage of NFC can remove the independent circuit and power supply in the BSN system, improving its durability. In addition to wireless sensing platforms, strain sensors with identification ability for emergency warning systems are also of great significance in real practical applications. We recently demonstrated an end-to-end health monitoring and emergency warning system in our previous work by integrating strain sensors with a convolutional neural network (CNN) (Fig. 10f) [153]. Human respiration signals were collected by attaching the strain sensor to the chest, and then the signals (i.e., normal breath, tachypnea, and tachypnea with cough) were classified into three target classes by using the CNN. The confusion matrix for the demonstration shows that the classification has a very high accuracy of 93.3%. The respiration monitoring and emergency warning system can be applied for the healthcare of covid-19-infected patients, which is of significance to relieve the stress on medical care personnel.

### Human–Machine Interaction and Entertainment

**Fig. 11:**
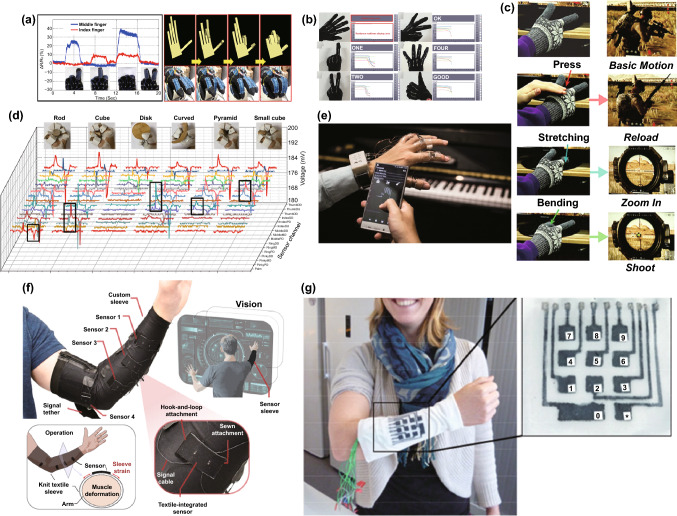
**a** The motion detection of two different fingers (left), and the mapping of fingers motions by strain sensor gloves (right) [166]. Copyright © 2014 American Chemical Society. **b** The gesture recognition by gloves with strain sensors [113]. Copyright © 2016 Wiley–VCH. **c** Controlling video games with smart gloves [210]. Copyright © 2019 Wiley–VCH. **d** Recognizing different grabbing gestures by feature outputs [211]. Copyright © 2020 American Association for the Advancement of Science (AAAS). **e** The optical image of the wireless instrument simulation system [127]. Copyright © 2017 Royal Society of Chemistry. **f** Photograph of a strain sensor-integrated sleeve for the future human–computer interfaces [212]. Copyright © 2020 Springer Nature. **g** The image of the fiber-based wearable keyboard [213]. Copyright © 2016 Wiley–VCH

The rapid development of flexible electronics has stimulated the application of artificial intelligence services, or high immersion technologies such as virtual reality (VR) and augmented reality (AR). These applications usually relate to human motions, while traditional electronic products like keyboards or mouses cannot be competent. In this situation, wearable strain sensors have become increasingly popular because of their flexibility and high performance. As early as 2012, hand movement monitoring through wearable strain sensors has already been mentioned, and an interlocking structure strain sensor with PU nanofibers on PDMS substrate was developed. By its sensitivity to shear (GF = 0.75), pressure (GF = 11.45), and torsion (GF = 8.53), the sensor arrays on the back of the hands can detect the movement of hands and the pressure on the sensor surface, illustrating the future of flexible strain sensors in interaction fields [4]. The demands of strain detection for large-deformation motion are considerable for many applications. A highly stretchable nanopaper/PDMS composite sensor was synthesized by Lee et al., where nanopapers were made by mixing nanocellulose and graphene [164]. By integrating five sensors responsible for different fingers, sensing gloves were prepared for hand motion detection. Benefitting from the high GF of 7.1 and the high stretchability of sensors, the resistances change can perfectly correspond to the bending of 5 fingers. Figure 11a shows a smart glove containing Ag NW sensors on fingers with a sandwich structure [166]. The research group successfully measured the position of different fingers more precisely by sensor resistance change with negligible latency. Moreover, the computer could display real-time images of finger motions by collection and communication system, emerging its possibility in complicated interactions like VR gaming or AR training.

The study of detection gestures with high accuracy has been continued for further optimization. Chen et al. combined fiber-based sensors with common gloves as a wearable interaction device [113]. The strain sensors, which have coated Ag NWs layer on PVDF fibers, illustrated outstanding sensitivity to monitor bending and strain. Researchers attached ten fiber sensors at interphalangeal joints and metacarpophalangeal joints to simulate gestures by analyzing the signal change from these ten joints. The sensor-computer system detected several gestures, such as numbers, “OK” and “good” with high feasibility (Fig. 11b). The ten sensitive sensors provide a high degree of freedom, and these data are meaningful for detecting complex hands motions comprehensively in interaction. In 2017, a skin-like strain sensor system was also reported to monitor the human's joint motions [208]. By coating rGO sheets on porous PDMS substrate, the sensor in this research demonstrates a reliable performance (GF = 7 at 10% strain and 173 at 40% strain). Sensors on the system were attached to knees and fingertips, enabling people to monitor different motions and frequencies on their phones. Outstanding biocompatibility for this device was also proved, which is quite essential for long-term wearing. Very recently, motion detection for both arm movements and hand gestures has also been achieved by using a wearable sensor system. Zhang et al. reported data gloves with ten high stretchable fiber-based strain sensors and sensor-assembled data bands, providing the capability of detecting motions of hands, wrist, elbows, and shoulders [209]. By these devices, researchers gathered human arm/hand gesture data and trained a neural network to recognize gestures. The system can recognize 20 alphabetic signs (in a total of 26 signs) with high accuracy of 93.33%. Arm gestures, especially dynamic police traffic gestures were monitored and recognized, illustrating its future in complicated motion interactions.

The applications for human–machine interaction and VR simulation were also studied. Figure 11c shows a fiber-based sensor system for gaming [210]. The fiber-based conductive strain sensors consist of two twisted PU-Ag NP fibers with hairy composite coating. The hairy microstructure successfully detected different stimuli such as pressure, stretching, and bending by different resistance change waveforms, which are significant for detecting complicated hand motions. The intelligent glove system, where two sensors were integrated into thumb and index fingers, was proved to immediately recognize different motions like pressing, bending, and stretching with high accuracy. Except for determining the hand motions by mapping resistance difference, different movements could also be detected by feature resistances of some parts of the hand. Zhu et al. designed smart gloves with wearable sensors and palm sliding sensors for comprehensive hand motion detection [211]. A triple-sections design for finger bending sensors was used, which projected the movement of fingers on a 30° scale by the virtual hand in Unity software successfully; researchers also achieved basic mouse control by the actions of different fingers. Additionally, the palm sensors can detect the normal force and shear force by detecting the amplitude of the negative triboelectric output and the opposite signal from different sections. This capability can assist researchers in recognizing gestures and actions more precisely. In identification tests, different grabbing actions and grabbed materials were recognized from distinct feature signals with an accuracy of 96% (Fig. 11d). In VR surgery simulation applications, different characteristic signals caused by different holding habits were successfully analyzed by gloves. The usage of gauze, scissors, and scalpels could also be simulated precisely, which provided a vivid interaction in VR gaming and virtual social network for users. Figure 11e exhibits a small-scale motion detector for fingers with graphene-modified fabric/PDMS composites piezoresistive strain sensors [127]. The interconnection structure and good adhesion between composites interface contribute to an orthotropic sensitivity, with more than 200% resistance change in 3% strain and a fast response time (0.072 s). When playing the piano, tiny muscle movements can be converted to unique signals by attached sensors at the fingers and wrist. Researchers corresponded different notes and chords to the feature signals and successfully played music by the strain sensors system and smartphones with the wireless connection between two devices. Very recently, a motion detection sleeve integrated with four wearable strain sensors was reported (Fig. 11f) [212]. Sensors in this system exhibit an outstanding linear response (*R*^2^ > 0.98), high sensitivity (GF > 9000), and low resistive change to off-axis deformation. Thanks to sensors’ high performance, the sleeve can detect the small muscle motions of the arm caused by hand movements. By neural network training, these movements of hand and wrist were successfully analyzed and traced, such as different gestures and the rotation of the wrist, even the metacarpophalangeal angle in distinct motions. The precise detection of this glove-free system opened a novel approach to complicated human–machine interactive interfaces and applications which need high hands mobility.

Apart from sensing the motions of hands and arms, some other interaction methods like typing and facial movements are also vital for users. Keyboards are still high-efficiency input tools in human–machine interaction, and textile-based sensors may become a competitive candidate because of their relatively low cost and high durability. Figure 11g exhibits a stretchable capacitive electrodes keyboard with horseshoe structure knitted fabric and PEDOT:PSS conductive polymer [213]. Up to 8 times capacitance change (less than 1–8 pF) can be measured when pressing by fingers. This high stretchable textile-based sensor array is suitable for outdoor wearing applications, such as interaction with devices when exercising. Facial motions are also significant in some special conditions. A textile-based sensor system that could detect users' speaking and blinking in real-time was reported [170]. The capacitive topographical-structure sensor in this system provides high sensitivity at small deformation. Thus, blinking and speaking were monitored as capacitance change by the motion-caused bending of sensors in this research. Language and head action interaction may be realized, which is significant for disabled or some patients. Besides, joint bending monitoring was studied in robot hands, exhibiting the sensor’s wide application potential. Most wearable strain sensor systems for human–machine interaction focused on hand motion since using hand is the most essential and efficient way for people to interact. Current sensor systems have already become accurate, illustrating their future in real applications. Apart from accuracy, durability, wearability, aesthetics, and other functions are still waiting for optimization in further study.

## Sensing Reliability and Function Integration

**Fig. 12:**
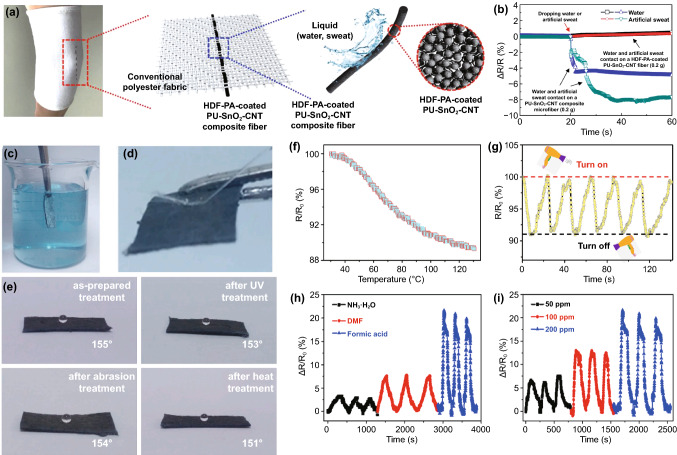
**a** Photograph and schematic diagram of the prepared composite microfiber seamed in the elbow wrap, the fabricated fiber could repel both artificial sweat and water. **b** Relative resistance changes of the composite microfibers with and without the modification of HDF-PA depending on sweat and water [214]. Copyright © 2018 Wiley–VCH. **c** Photo of the mirror-like phenomenon of the sensor by immersion in water and **d** a column of water bouncing off. **e** Photo image showing the WCA variation after different treatments [138]. Copyright © 2019 Elsevier. **f** The relative resistance change depends on temperature from 30 to 130 ℃. **g** The sensing behavior of the hot wind. **h** Cyclic sensing performance of different vapors under the concentration of 200 ppm. **i** Sensing performance of formic acid with different concentrations [218]. Copyright © 2020 Elsevier

The FWSSs can monitor human motion and health status in real-time. Because of the complicated microclimate on the skin surface, they may be influenced by sweat or water from the practical application environment and cause the loss of efficacy. Taking this kind of impact into consideration, Ju et al. developed a novel fiber strain sensor that focuses on the issue, and there is no additional protective layer on the outside of the sensor [214]. The author fabricated a HDF-PA-PU-SnO_2_-CNT composite microfiber strain sensor by conventional wet-spinning. The CNTs embedded in the PU matrix play a key role in monitoring the motion of the elbow by testing and recording the change of resistance. SnO_2_ coated by HDF-PA was embedded in PU to restrain water or sweat entering the fiber, allowing accurate detection of the elbow motions even when exposed to artificial sweat or water (Fig. 12a). As the CNT weight increases, both the contact angle of the prepared composite microfibers for sweat and pure water increases from 119.8°/119.4° to 144.2°/143.2°, which can be attributed to the increase of surface roughness with the adding of CNT. Figure 12b illustrates the relative resistance change of the fabricated composite fiber with and without HDP-PA modified when immersing in sweat and water. The resistance of the fiber without the treatment of HDF-PA decreases rapidly since sweat and water can penetrate the inner part of the microfiber and affect the conductivity. In addition to the interference of sweat and water, temperature is also a common influence factor. Based on this, Shen et al. presented a super-sensitive, wide strain range, fast response and durable strain sensor, consisting of carbon black/TPU and Ecoflex [215]. The strain sensor was fabricated by depositing carbon black (CB) NPs on the oriented TPU by ultrasonic treatment, with Ecoflex encapsulation on both top and bottom layers, forming a sandwich-like structure. The Ecoflex layer provides effective protection to the core part of the sensor, the conductive CB/TPU composite network, vesting the strain sensor an excellent anti-interference ability against external humidity and temperature. The resistance of the strain sensor shows little change with the humidity and temperature change. Under a small strain, the CB NPs anchored on the TPU surface can be separated in the direction of stretching, causing partial damage of conductive paths. As the strain increases, CB NPs are further separated, with a noticeable conductivity descent, until the CB NPs network becomes completely disconnected. Upon the strain released, CB NPs can return to their original positions, leading to the recovery of the resistance. Additionally, previous research outputs have investigated how the environmental factors affect the electromechanical properties of flexible strain sensors made with CNTs [216, 217], especially focusing on the temperature and relative humidity in determining sensing reliability. It is significant progress considering the attention to wearable microclimate, in contrast to the large neglect in previous research.

With the rapid development of wearable smart devices, there is an increasing demand for the strain sensor with combined functions, such as excellent waterproof performance, self-cleaning, and corrosion resistance. An adjustable micro-cracked non-woven fabrics strain sensor was developed based on cellulose nanocrystal/conductive graphene. Apart from the wide sensing range and high tactile sensitivity, the super hydrophobicity was also obtained due to hydrophobic fumed silica (Hf-SiO_2_) coating by additional dip-coating process [138]. The NWF-based sensor presents outstanding waterproofness, excellent self-cleaning, and stability. As seen in Fig. 12c, the NWF strain sensor shows a total reflectivity due to the air layer trapped on the microstructure of Hf-SiO_2_ surface, thereby efficiently protecting the NWF strain sensor from wetting. Water can bounce completely off the base surface with no residue (Fig. 12d). The author evaluated the anti-corrosion ability after different treatments to simulate the actual conditions in practical application (Fig. 12e). It indicates that the multifunctional strain sensor was successfully endowed with excellent hydrophobic via the simple dip-coating process in Hf-SiO_2_ suspension.

In addition to strain detection, a sensor with chemical vapor and temperature monitoring functions shows a broad vision of application in many fields, such as electronic skin, electrical nose, and thermal protection. Integrating all these multiple functions into a single flexible sensor remains a huge challenge. Very recently, an ultralight and soft sensor with polyamide 66 (PA66) nanofibers/conductive graphite nanosheet (GN) was produced by electrospinning and ultrasonic treatment [218]. The ultrafine GN/PA66 nanofiber shows the high aspect ratio characteristic, where the GN ensures an excellent heat transfer level. The synergistic effect guarantees that nanocomposites exhibit an obvious response to the temperature. Figure 12f shows the change of resistance with temperature varying from 30 to 130 ℃, attributing to the extrusion of the GN during the thermal expansion of nanofibers at higher temperatures. As shown in Fig. 12g, the resistance changes when the blower is turned on and turned off. Additionally, the porous structure vastly contributes to the gas sensing of the composite membrane. Considering that PA66 is a polar polymer, the author selected three polar chemical reagents as the vapor suppliers. Figure 12h illustrates that the GN/PA66 nanocomposite produces the highest resistance response in the formic acid vapor (22%) while providing the lowest resistance response of about 4% in the NH_3_·H_2_O vapor. A separate study for the gas-sensitive behavior of formic acid exhibited that the resistance response would strengthen as the vapor concentration increases (Fig. 12i). The multifunctional sensor can also detect different chemical vapors of DMF, formic acid, and NH_3_·H_2_O precisely regardless of the various bending states.

**Fig. 13:**
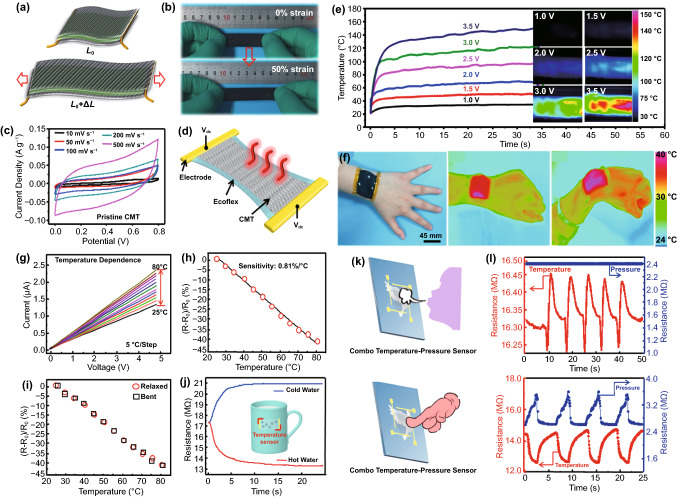
**a** Schematic of the stretchable CMT-based supercapacitor. **b–c** Photo image of the supercapacitor under the original state and strain of 50% and the current–voltage curves at different scan rates. **d** Schematic of stretchable CMT-based heater. **e** Time–temperature curves of the CMT heater under different voltages from 1 to 3.5 v. **f** A CMT heater affixed on a wrist and the corresponding IR images with the wrist under relax and bending state [144]. Copyright © 2017 American Chemical Society. **g–j** *I–V* curves, the relative resistance change with the temperature range from 25 to 80 ℃, and the resistance change with the temperature sensor under relaxed and bent conditions. **j** Resistance response to cold water and hot water. **k–l** Schematic illustration and the sensing performance of the E-skin under exhaling stimulus and finger pressing stimulus simultaneously [219]. Copyright © 2017 American Chemical Society

Zhang et al. developed a weft-knitted fabric based on carbon fibers as a supercapacitor and thermal-therapy device, which also exhibits stable performance when loading with tensile strains [144]. The high conductivity, porous structure, and inherent stretchability of carbonized Modal textile (CMT) ensure the excellent performance of the supercapacitors both in cyclic charge and discharge and stretchability. The CMT served as the active electrode of the all-solid, symmetric supercapacitor, with phosphoric acid-PVA gel as separator and electrolyte (Fig. 13a). It can be seen from Fig. 13b that the fabricated supercapacitor can be stretched up to 50% without damaging the structural integrity. Figure 13c demonstrates the CV curves of the CMT-based supercapacitor measured under different scan rates. The obtained CV curves present an equirectangular shape, illustrating the behavior of a typical double-layer capacitor. The structure of the stretchable CMT-based heater with Ecoflex encapsulation is illustrated in Fig. 13d. The CMT heater reached a stable temperature in ~ 4 s under a lower driving voltage in temperature pictures from IR camera (Fig. 13e), which is beneficial in practical applications. The author also fixed a CMT-based heater on the volunteer’s wrist to demonstrate its performance as a wearable heater. As shown in Fig. 13f, the CMT heater exhibits analogous temperature data, demonstrating the attractiveness of this inherently conductive and stretchable textile for wearable thermal-therapy devices.

By using a carbon fiber membrane derived from transparent silk nanofibers as an active material, a pressure–temperature composite e-skin was developed by combining a strain sensor and a temperature sensor [219]. The strain sensor exhibits ultra-high sensitivity at a strain of 50% (GF of up to 8350), which can detect tiny pressure stimuli causing local strain. The temperature sensor shows a high sensitivity of 0.81% per degree Celsius. To distinguish the detection of temperature and pressure simultaneously, the device was designed to respond independently. It can detect the spatial distribution of exhaling, finger pressing, pressure, and temperature. Figure 13g demonstrates the *I–V* curves of the temperature sensor over the temperature changes. Upon applying a fixed voltage, the current increases from 1.51 to 2.33 μA as the temperature increases from 35 to 80 ℃, indicating a significantly negative temperature coefficient. The sensitivity is higher than the relatively 0.81% per degree Celsius (Fig. 12h). Apart from the good stability, fast response and flexibility, the sensor also owns a constant response under both bent and relaxed states, which is of great significance in practical applications as e-skin (Fig. 13i). The author attached a sensor on a cup to reveal its temperature response (Fig. 13j). The resistance changed obviously when different-temperature water was poured into the cup, manifesting that the silk-based sensor could precisely measure the temperature change. The pressure and temperature detection property of the obtained combo e-skin was also demonstrated simultaneously by exhaling and finger pressing to simulate practical human activities (Fig. 13k). During the tests, the electrical resistance of the fabricated sensor dropped instantly when the volunteer exhaled to it, which occurred as a result of an increase in temperature caused by expiration. It then recovered to its initial value with the temperature decreasing, showing a cyclically stable signal to the periodic exhaling stimulus. Upon touching the sensor, the resistance decreased suddenly and then increased slowly to the initial level (red curve in Fig. 13l), indicating a good pressure sensing ability. Strain sensors with additional functions such as warming, cooling, sweat guiding, pressure, and temperature sensing are of great significance to practical applications, which should be intensely invested in the future. How to avoid the mutual interference among devices functions to ensure accuracy and independence is of vital importance.

## Conclusions and Perspectives

In summary, we have reviewed fiber-based wearable strain sensors with comprehensive investigation and detailed discussion. The classifications based on the measured physical quantification (i.e., resistive-type strain sensor, capacitive-type strain sensor, piezoelectric-type strain sensor, and triboelectric-type strain sensor) and their indicators for performance evaluation (i.e., sensing range, sensitivity, response and recovery time, linearity, hysteresis and cycling stability) of fiber-based strain sensors were introduced. Conductive fiber preparation for the fabrication of strain sensors from spinning, surface modification, and structural transformation was presented with examples, where a detailed discussion of the merits and demerits of all these methods was also given. We classified the devices into encapsulated and encapsulation-free strain sensors according to whether the encapsulated layer’s existence and then critically analyzed the advantages and disadvantages between them. The sensing mechanisms, such as crack formation, inherent changes of sensing components, disconnection effect, and geometry alteration, were expounded with examples of representative devices from two types, i.e., elastomer-encapsulated and encapsulation-free strain sensors. We also summarized and evaluated the potential of the fiber-based strain sensors in the applications of body sensing networks, multifunctional sensing, as well as human–machine interaction and entertainment.

Though considerable headway has been made in terms of the preparation approaches, performance optimization, and application widening of fiber-based strain sensors, some fields are still worth exploring. A grand challenge is performance improvement. Much research in recent years has focused on increasing the stretchability and sensitivity of strain sensors, and dramatic progress has been made to date. A strain sensor with high stretchability or remarkable sensitivity is not technically challenging, which can be achieved by combining electrical performance-different materials to construct multi-layer sensing elements. However, more efforts should be contributed to developing a strain sensor with both high sensitivity and a wide sensing range. The wide sensing range is more necessary than the sensitivity improvement, as the sensor signals can be easily amplified through specific electric apparatus in most cases. On the other hand, the linear relationship between strain deformation and electrical signal outputs is essential for strain sensors. Some studies reported strain sensors with the linear detecting ability within a certain degree of elongation range; however, the linearity throughout a large sensing range, the mechanism exploration, and verification behind the phenomenon still need to be studied further in the future. More importantly, other vital electromechanical properties such as hysteresis, stability, and durability indicate the detection accuracy and lifetime, which have been largely overlooked. Strain sensors should possess low hysteresis, outstanding stability, and durability, needing the development of low viscoelasticity and high robustness conductive materials. In addition, most properties of reported strain sensors were evaluated in the laboratories under certain conditions, while the electrical responses may be changed in real practical applications when surroundings with fluctuant temperature and humidity, or multiple-direction stress and deformations. The evolutions should be standardized in simulated wearable occasions in the future.

Wearability is another crucial evaluation index of wearable sensors, mainly including two aspects: how stable and flexible the sensor is when attaching to the moving surface; how comfortable and biocompatible the wearer is when wearing the sensor. The surface-mount ability and flexibility of strain sensors is the precondition in determining its reliability, which dramatically impacts the signals converted from the device. Although some sensors can be attached to the skin surfaces through prepared microstructures, the adhesion is still not desirable under different-range and multi-dimensional deformations. The interfaces between fiber/yarn/fabric and the skin determine the wearing comfort. The involuntary physical breathing of the human body constantly produces sweat from the skin surface to the environment by water vapor, and the breathing would be more active in doing sports. The interfaces can essentially influence the efficiency of vapor transport. Thus, strain sensors should possess good permeability to air, water moisture, and water vapor to expel body metabolites and guarantee a pleasant microenvironment between the sensor and the skin. The lack of breathability will lead to discomfort and even skin irritation. This index is of vital importance for wide-area body detection, such as the construction of full-range body sensing networks. Strain sensors with the encapsulation of elastomers to render good stability often have to deteriorate their breathability and wearability, which have not been paid enough attention. In addition to the wearing comfort, good biocompatibility of on-skin and implantable strain sensors is also crucial in practical applications. It avoids the harms caused by immune rejection, which is of great significance in long-term detection.

Strain sensors with anti-jamming and other auxiliary functions are also very desirable for wearable applications. Such tactile sensing devices detect surface motions by converting movements into electrical signals, while most of the surface motions at the time of strain deformation also include combination inputs such as twisting, pressuring, and bending. Nonetheless, a vast majority of reported strain sensors show significant responses to many deformations. It is very challenging to precisely sense motions in wearable occasions with the effects, especially in complex moving conditions. Therefore, strain sensors with the anti-jamming property to the mechanical inputs are of vital importance to accurately perceive motions in real-time. On the other hand, the microclimate in wearable interfaces always involves fickle temperature, humidity, and potential of hydrogen, which may significantly deteriorate sensor reliability considering some active materials are sensitive to the stimulation of wearable microclimate. Strain sensors should also maintain anti-jamming performance to the wearable microenvironment, without the concern of possible effects on sensing accuracy. Considering the applications of strain sensors as e-skin, endowing them with auxiliary functions such as thermal conductance, moisture transmissibility, warming, and cooling ability is also of great significance, which should be investigated in the future.

Moreover, the most current strain sensors need outside power and metallic interconnect to other electronic equipment for data collection and analysis. The tentative exploration proves that it is feasible for the sensors to harvest energy from the environment and the human body to power themselves. Compared with the wiring integration with poor monitoring continuity and hampered physical activity, wireless networks exhibit superior advantages in practical applications. It is worth noting that the high-performance, self-powered strain sensors need to be rapidly developed, realizing the monitor with long-term and unrestricted range of activity. Apart from that, more functions should be integrated into the platform and fiber-based sensor to build a smart and interactive wearable system. Functions such as display, camouflage ability, or even data storage/processing, or features that rely on the fiber performance like structural coloration and electroluminescence, are also vital for the smart system to realize the autonomous and intelligent multifunction. Strain sensors with the abovementioned functions would radically change the scenes and improve the quality of people's lives. We believe these challenges and opportunities for developing strain sensors need more interdisciplinary collaborations to push this thriving field forward.
